# Organoid-derived C-Kit^+^/SSEA4^−^ human retinal progenitor cells promote a protective retinal microenvironment during transplantation in rodents

**DOI:** 10.1038/s41467-019-08961-0

**Published:** 2019-03-14

**Authors:** Ting Zou, Lixiong Gao, Yuxiao Zeng, Qiyou Li, Yijian Li, Siyu Chen, Xisu Hu, Xi Chen, Caiyun Fu, Haiwei Xu, Zheng Qin Yin

**Affiliations:** 1 Southwest Hospital/Southwest Eye Hospital, Third Military Medical University (Army Medical University), Chongqing, 400038 China; 2 Key Lab of Visual Damage and Regeneration & Restoration of Chongqing, Chongqing, 400038 China

## Abstract

Stem cell therapy may replace lost photoreceptors and preserve residual photoreceptors during retinal degeneration (RD). Unfortunately, the degenerative microenvironment compromises the fate of grafted cells, demanding supplementary strategies for microenvironment regulation. Donor cells with both proper regeneration capability and intrinsic ability to improve microenvironment are highly desired. Here, we use cell surface markers (C-Kit^+^/SSEA4^−^) to effectively eliminate tumorigenic embryonic cells and enrich retinal progenitor cells (RPCs) from human embryonic stem cell (hESC)-derived retinal organoids, which, following subretinal transplantation into RD models of rats and mice, significantly improve vision and preserve the retinal structure. We characterize the pattern of integration and materials transfer following transplantation, which likely contribute to the rescued photoreceptors. Moreover, C-Kit^+^/SSEA4^−^ cells suppress microglial activation, gliosis and the production of inflammatory mediators, thereby providing a healthier host microenvironment for the grafted cells and delaying RD. Therefore, C-Kit^+^/SSEA4^−^ cells from hESC-derived retinal organoids are a promising therapeutic cell source.

## Abstract (web summary)

Stem cell transplantation to treat retinal degeneration could be limited by the degenerative microenvironment. Here, the authors show that C-Kit^+^/SSEA4^–^ progenitor cells enriched from human embryonic stem cell derived retinal organoids protect retinal structure, suppress microglial activation, gliosis and inflammation.

## Introduction

Retinal degeneration (RD) refers to a group of devastating blinding retinal disorders that share a common pathological process—the progressive loss of photoreceptors^1^. Currently, effective therapy for RD is lacking, and several alternative strategies are under investigation^2^. Among these strategies, stem cell transplantation is particularly promising; even at late stages of the disease, the transplanted cells can potentially replace dying photoreceptors and preserve vision. In addition, the eye is likely the most suitable organ for cell therapy due to its high immune privilege, the availability of relatively safe and easy surgical procedures, and the availability of noninvasive imaging and electrophysiological techniques to evaluate the outcome^3^. To date, several stem cell-based clinical trials have been conducted with RD patients^4^. However, the optimal cell source for transplantation remains elusive, which is one of the major obstacles in stem cell therapy of RD.

One promising donor cell source is retinal progenitor cells (RPCs)—retina-specific stem cells that are capable of self-renewal and differentiation into various retinal cell types. Human RPCs (hRPCs) derived from human fetal retinas^5,6^ have been shown to preserve visual function when transplanted into the subretinal space (SRS) of Royal College of Surgeons (RCS) rats^7^. In a series of clinical trials, intravitreal and subretinal injections of hRPCs were performed in retinitis pigmentosa patients for safety and tolerability evaluation^4,8^. However, the use of human fetal retinas is restricted by availability and ethical issues. Alternatively, human embryonic stem cells (hESCs) can be induced in vitro to form 3D retinal organoids^9,10^ from which donor cells can be harvested. This method allows cell expansion and manipulation in vitro with low variability, which is critical for clinical standardization and industrialization. Inspiringly, previous studies have shown that photoreceptor precursor cells (PPCs) or retinal pigment epithelium (RPE) derived from ESC-derived retinal organoids demonstrated a mature structure and superb function^11,12^. However, isolating RPCs from hESC-derived retinal organoids (hEROs) while avoiding contamination with undifferentiated ESCs remains a key challenge in stem cell therapy.

Thus, cell surface markers are of particular clinical significance for enriching donor cells. Surface antigen C-Kit, also known as CD117, is a type III receptor tyrosine kinase that binds to stem cell factor (SCF) and was previously found expressed in several types of stem cells such as hematopoietic stem cells and spermatogonial stem cells^13,14^. Previous studies have consistently demonstrated that C-Kit marks a population of RPCs in developing mouse and human retinas and is thus a promising candidate for screening of hRPCs^15–17^. Another cell surface marker, stage-specific human embryonic antigen-4 (SSEA-4, SSEA-1 in mice), is expressed at the early stage of embryonic development and might be useful for identifying and eliminating cells of embryonic origin that are potentially tumorigenic^18^. Indeed, previous studies found that isolated C-Kit positive and SSEA-1/4 negative cells (C-Kit^+^/SSEA-1/4^−^ cells) from both mouse and human fetal retinas possessed the characteristics of RPCs and were capable of rescuing the vision of RD animals after transplantation^16,17^. Therefore, it will be of great therapeutic interest to investigate whether we can enrich C-Kit^+^/SSEA4^−^ hRPCs from hEROs and to determine whether they are an optimal donor cell source for transplantation.

The efficacy of cell transplantation, especially transplantation for extended periods, depends not only on the intrinsic properties of the donor cells but also on the microenvironment of the host tissue^19,20^. In degenerative retinas, reactive microglia often cause neuroinflammation, which is unfavorable for long-term survival and proper differentiation of grafted stem cells^19^. It was shown that additional strategies for improving the microenvironment need to be combined with stem cell transplantation to achieve better therapeutic outcomes^21^. However, it would be ideal to obtain donor cells that possess both RPC characteristics and the ability to improve the host environment. Interestingly, C-Kit and its ligand SCF have been reported to play a key role in microglial modulation^22,23^. However, whether C-Kit^+^/SSEA4^−^ cells from hEROs can also modulate microglia is not clear. Exploring this question may significantly improve transplantation outcomes.

Here, we report that C-Kit^+^/SSEA4^−^ cells enriched from hEROs are indeed RPCs that are non-tumorigenic and highly similar in transcriptional profile to native hRPCs from human fetal retina. Moreover, when transplanted, C-Kit^+^/SSEA4^−^ cells demonstrate a unique ability to improve the unfavorable microenvironment present in the degenerative retina and thus can achieve superb survival, proper differentiation and incorporation. Consequently, transplantation of C-Kit^+^/SSEA4^−^ cells significantly preserves the visual function of RCS rats for as long as 24 weeks. In summary, we establish a feasible strategy for acquiring optimal donor cells for transplantation and shed light on stem cell therapy in RD.

## Results

### Spatiotemporal expression of C-Kit and SSEA4 in hEROs

**Fig. 1:**
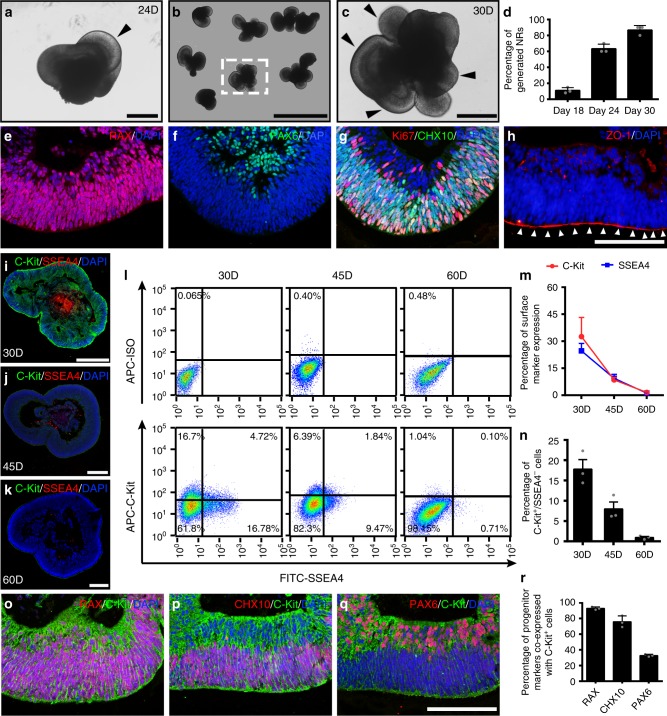
Identification and C-Kit/SSEA4 expression in hEROs. **a** Induction of the 24-day neural retina structure (black arrowhead). **b** Induction of the 30-day neural retina. **c** Magnification of the neural retina (black arrowheads) shown in **b**. **d** Quantitative analysis showing that the number of generated neural retinas increased with induction time (18-day: 11.1 ± 1.2%; 24-day: 63.3 ± 1.9%; 30-day: 86.7 ± 1.9%; *n* = 3 independent experiments/group; each experiment containing 96 hEROs.). **e**–**g** Expression of the RPC markers RAX, PAX6, and CHX10 and the proliferation marker Ki67 in hEROs. **h** Identification of the apical side of hEROs on day 30 via ZO-1 staining. **i**–**k** Distribution of C-Kit and SSEA4 antigens in 30-day, 45-day, and 60-day hEROs. **l** Flow cytometry analysis of C-Kit and SSEA4 expression in hEROs at different induction times (30-day, 45-day, and 60-day). **m** Statistical analysis of C-Kit and SSEA4 expression in hEROs (C-Kit: 30-day: 32.60 ± 10.58%; 45-day: 8.53 ± 1.36%; 60-day: 1.58 ± 0.45%; SSEA-4: 30-day: 24.6 ± 4.16%; 45-day: 9.35 ± 2.23%; 60-day: 1.17 ± 0.88%, *n* = 3 independent experiments/group; each experiment containing 192 hEROs). **n** Percentage of C-Kit^+^/SSEA4^−^ cells determined by flow cytometry analysis (30-day: 17.80 ± 4.06%; 45-day: 7.99 ± 2.96%; 60-day: 0.96 ± 0.42%; *n* = 3 independent experiments/group; each experiment containing 192 hEROs). **o**–**q** Co-expression of C-Kit and RPC markers in 30-day hEROs. **r** Quantitative analysis of RPC marker expression in C-Kit^+^/SSEA4^−^ cells in 30-day hEROs (RAX: 92.86 ± 1.88%; CHX10: 75.79 ± 7.46%; PAX6: 32.74 ± 1.58%; *n* = 3 independent experiments/group; each experiment containing 3 hEROs). Data are presented as mean ± SD. Scale bars, 200 μm (**a**, **c**, **i**–**k**), 1 mm (**b**), 50 μm (**e**–**h**, **o**–**q**). hEROs hESC-derived retinal organoids

To investigate whether it is feasible to enrich RPCs from hEROs using the surface marker C-Kit/SSEA4 and to determine the optimal time window for cell harvesting, we first characterized the spatiotemporal profile of C-Kit and SSEA4 expression in hEROs. Neural retina structures could be initially identified at a low percentage of 11.1 ± 1.2% beginning at approximately day 18. Neural retina structures became prominent and larger by day 24 (Fig. 1a), and the positive rate of generated neural retinas reached 90% at day 30 (Fig. 1b–d). Neural retinas were evaluated from day 18 to day 60 to determine the expression patterns of retina-specific markers (Supplementary Fig. 1a–l). The RPC markers RAX, PAX6, and CHX10 and the proliferation marker Ki67 were observed in hEROs at day 30 (Fig. 1e–g, Supplementary Fig. 1b, f). The apical-basal polarity of the neural retina was defined by ZO-1 staining at the apical side (Fig. 1h). Spatially, C-Kit^+^/SSEA4^−^ cells were located in both the outer and inner layers of the hEROs at day 30 (Fig. 1i). With culturing, the expression of C-Kit decreased and was limited to the inner layer at day 45 and day 60 (Fig. 1j, k). Conversely, the embryonic marker SSEA4 was mainly found in the centers of the retinal organoids and decreased with culturing (Fig. 1i–k). Using flow cytometry (FCM), we found that C-Kit expression decreased gradually; the expression of SSEA4 presented a similar, albeit steeper, decreasing trend (Fig. 1l, m). We estimated the percentage of C-Kit^+^/SSEA4^−^ cells in hEROs to be 17.80 ± 4.06%, 7.99 ± 2.96% and 0.96 ± 0.42% at day 30, day 45 and day 60, respectively (Fig. 1n). We performed double-labeling of C-Kit and retinal progenitor markers in hEROs at day 30 and showed that C-Kit co-expressed with RAX, CHX10 and PAX6 (92.86 ± 1.88%, 75.79 ± 7.46%, and 32.74 ± 1.58%) respectively (Fig. 1o–r), supporting the idea that C-Kit marks a population of RPCs. Thus, we established a foundation for acquiring appropriate donor cells by successfully generating hEROs and characterizing the spatiotemporal expression pattern of C-Kit.

### Gene expression patterns of C-Kit^+^ cells and primary hRPCs

**Fig. 2:**
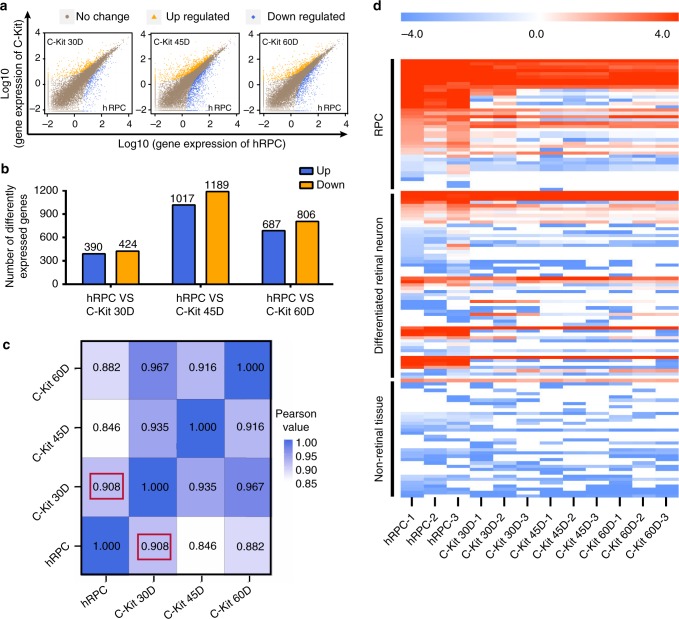
Transcriptome analysis of C-Kit^+^ cells from hEROs and primary hRPCs. **a**, **b** Transcriptome analysis showing differentially expressed genes and quantitative analysis of gene expression in primary hRPCs and in C-Kit^+^ cells from hEROs at different induction times (*n* = 3 biologically independent samples/group). **c** Pearson correlation analysis of all gene expression in hRPCs and in C-Kit^+^ cells from hEROs at different induction times (C-Kit-30D vs hRPC: 0.908 marked by the red box, C-Kit-45D vs hRPC: 0.846, C-Kit-60D vs hRPC: 0.882; *n* = 3 biologically independent samples/group). **d** Heatmap analysis of specific gene regions including RPCs, differentiated retinal neurons and non-retinal tissues as listed in Supplementary Data 1. Log_2_ FPKM expression levels of the genes are shown as a red-white-blue gradient. FPKM averaged fragments per kilobase of exon per million fragments mapped

To further characterize the cell features, we isolated C-Kit^+^/SSEA4^−^ cells (herein referred to as C-Kit^+^ cells) from 30-, 45-, and 60-day hEROs via fluorescence-activated cell sorting (FACS) and compared the isolated cells with primary hRPCs obtained from 12- to 14-w human fetal retinas at the transcriptome level. C-Kit^+^ cells from 30-, 45-, and 60-day hEROs had 814, 2206, and 1493 differentially expressed genes (DEGs), respectively, at the level of two-fold or greater difference (Fig. 2a, b). The correlation coefficients of all genes expression levels between those cells and hRPCs were very high (0.908, 0.846, and 0.882, respectively; Fig. 2c). We further analyzed the gene expression patterns of retina-specific genes and non-retinal genes (non-retinal central nervous system, hepatic, cardiac, renal and hepatic genes)^24^ and found that C-Kit^+^ cells exhibit a gene expression pattern similar to that of primary hRPCs, with high expression levels of RPC genes and differentiated retinal cell genes and very low expression of non-retinal tissue genes (Fig. 2d). Notable, C-Kit^+^ cells from 30-day hEROs showed the highest correlation in gene expression with hRPCs and the most similar expression pattern to hRPCs. Based on this finding and on the temporal expression pattern of C-Kit in hEROs (Fig. 1), we chose to use C-Kit^+^ cells from 30-day hEROs in the following experiments.

### C-Kit^+^ cells from hEROs are a non-tumorigenic pool of RPCs

**Fig. 3:**
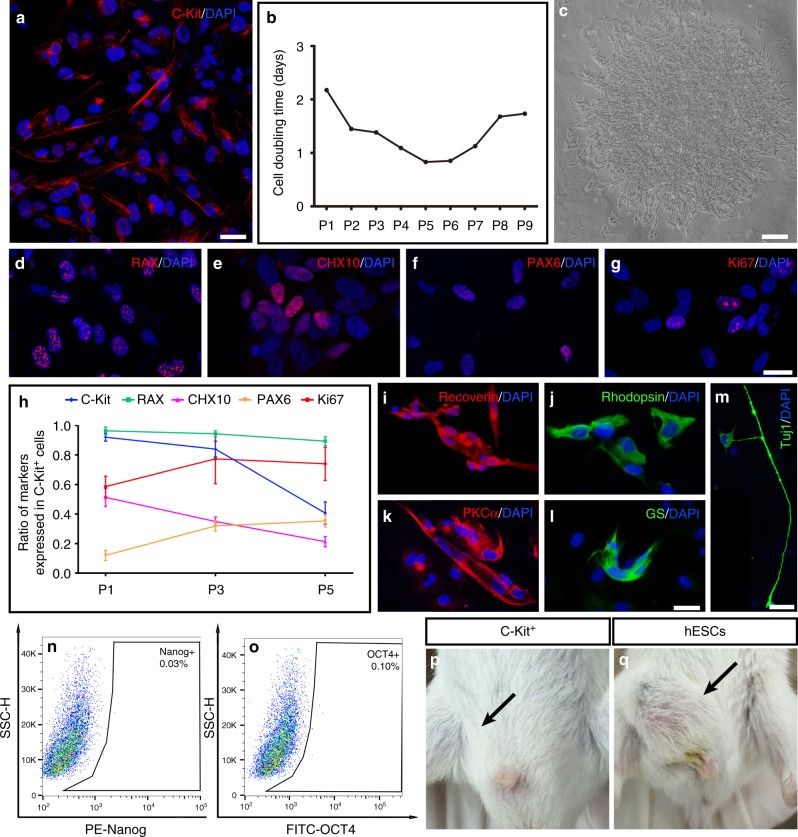
Characteristics of C-Kit^+^ cells obtained from hEROs. **a** C-Kit expression in 30-day sorted C-Kit^+^ cells at passage 0 (P0). **b** Comparison of cell doubling time among C-Kit^+^ cells sorted from 30-day hEROs (*n* = 3 independent experiments/group). **c** Representative putative clone formation by P3 C-Kit^+^ cells at 2 weeks shown through the limited dilution colony-forming efficiency assay. **d**–**h** Expression and quantitative analysis of RPC markers and the proliferation marker Ki67 in C-Kit^+^ cells at different passages. (*n* = 3 independent experiments/group). **i**–**m** Retinal cell differentiation of C-Kit^+^ cells into photoreceptors was visualized by immunostaining for recoverin and rhodopsin. Bipolar cells were identified by PKCα staining, Müller cells were identified by GS staining, and retinal ganglion cells were identified by Tuj1 staining. **n**, **o** Flow cytometry analysis of the expression of embryonic markers, including Nanog and OCT4, in P3 C-Kit^+^ cells. (*n* = 3 independent experiments). **p**, **q** Tumorigenesis test of P3 C-Kit^+^ cells and human embryonic stem cells (hESCs). No tumor formation was detected in the inguinal region 3 months after C-Kit^+^ cell injection into 6 SCID mice (black arrow in **p**). After hESCs injection, 4/6 animals displayed tumor formation (black arrow in **q**) (*n* = 6 animals/group). Data are presented as mean ± SD. Scale bars, 25 μm (**a**, **d**–**g**, **i**–**m**), 400 μm (**c**)

We sorted C-Kit^+^ cells from 30-day hEROs and re-probed C-Kit expression after sorting. The percentage of C-Kit expression in C-Kit^+^ cells at passage 0 (P0) was 92 ± 2.6% (Fig. 3a). C-Kit^+^ cells were cultured as a monolayer in 3% O_2_ because hypoxia promotes the proliferation and expansion of RPCs (Supplementary Fig. 2a–j)^25^. The cell doubling time was used to measure the proliferation rate of C-Kit^+^ cells, which peaked between P3 and P7 (Fig. 3b). We demonstrated clone formation from a single P3 C-Kit cell, further verifying the self-renewal capability of C-Kit^+^ cells (Fig. 3c). To probe how C-Kit^+^ cells evolve as they are passaged, we examined the expression of the RPC markers RAX, CHX10, and PAX6 (Fig. 3d–f) and that of the proliferation marker Ki67 (Fig. 3g) in P3 C-Kit^+^ cells enriched from 30-day hEROs. As in the hERO cultures, C-Kit expression decreased as the cells were passaged, decreasing from ~80% at P3 to <50% at P5. Among the RPC markers, RAX expression was maintained at a high level of 90%. PAX6 and Ki67 expression increased slightly, while CHX10 expression decreased from P1 to P5 (Fig. 3h). To examine the RPC features of the C-Kit^+^ cells, we applied a photoreceptor- specific differentiation protocol^16^ to P3 C-Kit^+^ cells. After application of this protocol, the cells expressed both recoverin and rhodopsin (Fig. 3i, j). Additionally, we used another retinal cell differentiation protocol; in that case, the cells differentiated into putative bipolar, Müller, and ganglion cells, as marked by PKCα, GS, and Tuj1, respectively (Fig. 3k–m). Furthermore, we confirmed that C-Kit^+^ cells were negative for the embryonic markers Nanog and OCT4, a result that suggests that cells of tumorigenic origin were successfully eliminated (Fig. 3n, o). Consistently, a tumorigenesis test revealed the absence of tumor formation following C-Kit^+^ cell injection into SCID mice with hESCs as a positive control (Fig. 3p, q). In summary, we verified that C-Kit^+^ cells are a population of RPCs of non-tumorigenic origin, and we chose P3 as our source cells for transplantation based on its relatively stable C-Kit expression, RPC features and cell viability.

### Therapeutic effect of C-Kit^+^ cells on RCS rats

**Fig. 4:**
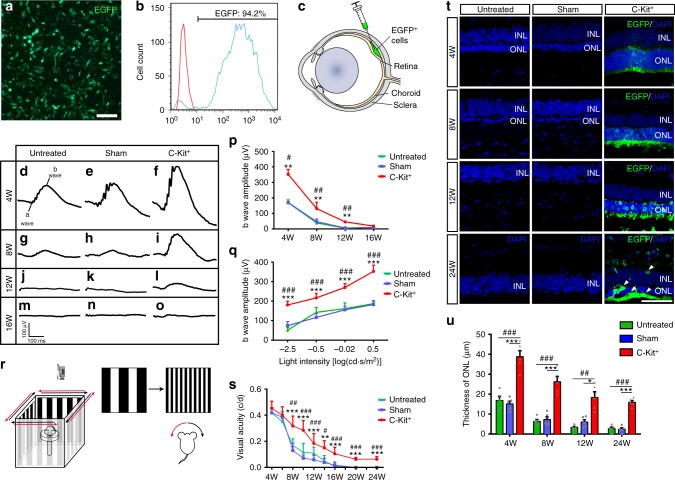
Protective effect of grafted C-Kit^+^ cells on RCS rats. **a** Identification of P3 EGFP hERO-derived cells transduced with lentivirus. **b** Flow cytometry analysis showing the EGFP^+^ rate of P3 hERO-derived cells. **c** Diagram showing the subretinal transplantation. **d**–**o** Representative b-waves of the C-Kit^+^, sham, and untreated groups at PO 4w, 8w, 12w, and 16w measured through scotopic flash electroretinography (fERG) at 0.5 log(cd*s/m^2^). **p** Statistical analysis of the amplitudes of ERG b-waves at 0.5 log(cd*s/m^2^) in the three groups from PO 4–16w. *n* = 5 eyes/group. **q** ERG b-waves of different light intensity stimuli at PO 4w. *n* = 5 eyes/group. **r** Diagram showing the setup of the optokinetic response test. **s** Visual acuity analysis obtained through the optokinetic response test at various post-operational times up to 24 weeks. *n* = 6 eyes/group. **t** Representative images of the ONL in the three groups from PO 4–24w. EGFP^+^ cells showing surviving cells at PO 24w (white arrowheads). **u** Statistical analysis of ONL thickness in the three groups at four time points (*n* = 5 eyes/group). *P* values were determined by one-way analysis of variance (ANOVA) test followed by Tukey (equal variances) or Dunnett’s T3 (unequal variances) multiple comparison tests **p**, **q**, **s**, **u** C-Kit^+^ cells vs Sham: *P < 0.05, **P < 0.01, ***P < 0.001; C-Kit^+^ cells vs Untreated: ^#^*P* < 0.05, ^##^*P* < 0.01, ^###^*P* < 0.001. Data are presented as mean ± SEM. Scale bars, 100 μm (**a**), 50 μm (**t**). ONL outer nuclear layer, INL inner nuclear layer

Next, we tested the safety and therapeutic effects of hERO-derived RPCs upon subretinal transplantation in postnatal day 21 (P21) RCS rats, a classic model of RD. To trace the donor cells, we transfected the donor cells with CMV::EGFP-lentiviral vectors, which labeled more than 94% of the donor cells (Fig. 4a, b). We performed transplantations of three groups of cells: (1) unsorted cells, (2) C-Kit^+^ cells (C-Kit^+^/SSEA4^−^ cells) and (3) C-Kit^−^/SSEA4^−^ cells (herein referred to as C-Kit^−^ cells) from 30-day hEROs into RCS rats (Fig. 4c). Notably, abnormal cell proliferation and tumor-like masses were observed in 4 of 6 RCS rats transplanted with unsorted cells at post-operational 4 weeks (PO 4w); these masses contained SSEA4 and Ki67 positive cells (Supplementary Fig. 3a–i). In contrast, neither cell proliferation nor tumor-like masses were observed in any of more than 50 RCS rats that received transplants of C-Kit^+^ cells up to PO 24w. Furthermore, we found that among an unsorted cell suspension obtained from 30-day hEROs, the percentage of SSEA4^+^/C-Kit± cells (herein referred to as SSEA4^+^ cells) was up to 24% (Supplementary Fig. 3j). Taken together, these observations suggest that negative selection for SSEA4 might be an effective way to eliminate undifferentiated cells that are at an early developmental stage and thus decrease the risk of tumor formation following transplantation.

We further performed scotopic flash electroretinography (fERG) in the C-Kit^+^ and C-Kit^−^ cell-transplanted groups to determine their treatment outcomes. The fERG test showed that transplantation of C-Kit^+^ cells in RCS rats significantly increased the amplitude of b-waves compared with the sham and untreated groups at PO 4w, PO 8w, and PO 12w (Fig. 4d–l, p), although the effect was less pronounced at PO 16w (Fig. 4m–o, p). Moreover, significantly enhanced b waves were also observed in the PO 4w C-Kit^+^ group at a wide range of light intensities (Fig. 4q). Additionally, RCS rats that received transplanted C-Kit^+^ cells demonstrated better vision preservation than rats that received C-Kit^−^ cells as shown by fERG at both PO 4w and 8w (Supplementary Fig. 3k).

Using the optokinetic response (OKR) test, a more sensitive method, we evaluated the animals’ visual acuity from PO 4w to PO 24w (Fig. 4r). In contrast to the sham and untreated groups, in which visual acuity sharply declined beginning at PO 6w and continuing through PO 24w, the C-Kit^+^ group performed significantly better and even stabilized throughout the period up to PO 24w (Fig. 4s). Morphologically, we found that grafted C-Kit^+^ cells significantly preserved the photoreceptors from loss at PO 4w, PO 8w, PO 12w, and PO 24w (Fig. 4t, u). Inspiringly, we observed that many C-Kit^+^ cells survived up to PO 24w (white arrowheads, Fig. 4t), demonstrating robust survival ability and promising potential for achieving relative long-term efficacy in clinical applications.

### Integration and material transfer of grafted C-Kit^+^ cells

**Fig. 5:**
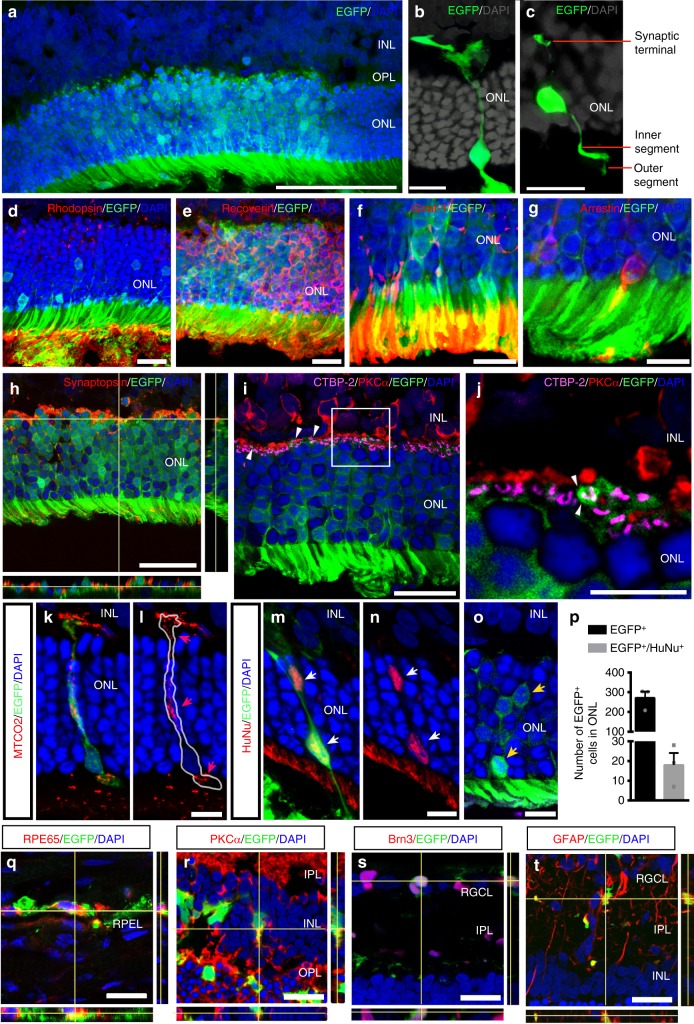
Integration and materials exchange of grafted C-Kit^+^ cells. **a** EGFP^+^ cells in ONL of the RCS rat retina at PO 4w. **b**, **c** EGFP^+^ cells showing typical photoreceptor morphology in outer segment at PO 4w. **d**, **e** Photoreceptor marker identification demonstrated by immunostaining for rhodopsin and recoverin at PO 4w. **f**, **g** Outer segment staining of rod Gnat-1 and cone arrestin at PO 4w. **h** Orthogonal projection of synaptophysin staining between EGFP^+^ cells and host inner retina at PO 4w. **i**, **j** EGFP^+^photoreceptor terminal (small white arrowhead) touching synaptic protein CTBP-2 and lying in close proximity to bipolar cells with PKCα staining at PO 4w. **k**–**n** EGFP^+^ cells in the ONL co-stained with a human-specific MTCO2 mitochondrial antibody (red arrowheads) (**k**, **l**) and a human-specific HuNu nuclear antibody (white arrowheads) (**m**, **n**), confirming the human origin. **o** EGFP^+^ cells in the ONL without MTCO2 or HuNu co-staining (yellow arrowheads). **p** Statistical analysis of the number of the EGFP^+^ and EGFP^+^/HuNu^−^ cells in the ONL. 6 ± 2% EGFP^+^/HuNu^+^ cells and 94 ± 5% EGFP^+^/HuNu^−^ cells in ONL (*n* = 814 cells from three eyes). **q**–**t** EGFP^+^ cells at PO 8w showing co-expression with the retinal pigment epithelial cell marker RPE65 (**q**), the bipolar cell marker PKCα (**r**), the retinal ganglion cell marker Brn3a (**s**) and the Müller cell marker GFAP (**t**). Data are presented as mean ± SEM. Scale bars, 100 μm (**a**), 20 μm (**h**, **i**, **q**–**t**), 10 μm (**b**–**g**, **j**–**o**). RGCL retinal ganglion cell layer, IPL inner plexiform layer, OPL outer plexiform layer, RPEL retinal pigment epithelium layer

The idea that grafted cells have the potential to differentiate into retinal cells and provide replacement therapy was a previously held belief^26^. However, recent studies have shown that the majority of predicted integrated photoreceptors were the result of cellular exchange, and such exchange is likely to be a more efficient approach to rescuing degenerative cells^27–29^. In the present study, clusters of EGFP^+^ cells were identified in the outer nuclear layer (ONL) at PO 4w (Fig. 5a). In cases of solitary EGFP^+^ cells, they presented the typical morphology of cone (Fig. 5b) or rod photoreceptors (Fig. 5c). Many of the EGFP^+^ photoreceptor-like cells expressed photoreceptor-specific markers such as recoverin and rhodopsin (Fig. 5d, e). Moreover, at the outer segment end, markers for rods (Gnat-1) and cones (cone arrestin) were detected (Fig. 5f, g), and at the synaptic terminal end, synaptophysin (a presynaptic protein) was expressed (Fig. 5h). In addition, the EGFP^+^ cells appeared to form ribbon synapses (marked by CTBP-2) with host rod bipolar cells (marked by PKCα) (Fig. 5i, j). Because we transplanted human C-Kit^+^ cells into host rat retinas, the grafted cells could be distinguished from the host cells. We used antibodies against human mitochondria (MTCO2) and human nuclei (HuNu) to identify the EGFP^+^/MTCO2^+^ photoreceptor-like cells (Fig. 5k, l, red arrowheads) and EGFP^+^/HuNu^+^ cells (Fig. 5m, n, white arrowheads) of human origin, which are likely to be the truly integrated cells. The results showed that some EGFP^+^/HuNu^+^ cells also express photoreceptor markers such as recoverin and Gnat-1 (Supplementary Fig. 4a–f). In contrast, clusters of EGFP^+^/MTCO2^−^ or EGFP^+^/HuNu^−^ cells (Fig. 5o, yellow arrowheads) are likely to represent host cells that acquired EGFP through material exchange. In our quantitative analysis, 6 ± 2% EGFP^+^/HuNu^+^ (18 ± 3.5) cells were observed in the ONL, while 94 ± 5% of the EGFP^+^ cells did not co-localize with HuNu (253 ± 19) (Fig. 5p) (mean ± standard error of the mean (SEM), *n* = 814 cells from three eyes). These findings demonstrate that both integration (albeit in a small population) and material exchange occurred following C-Kit^+^ cell transplantation. Interestingly, the integration or material exchange might depend on donor cell type, as we transplanted C-Kit^+^ cells, which are distinguished from photoreceptor precursors by having the potential to differentiate into multiple retinal cell types. At PO 8w, some EGFP^+^ cells in the SRS co-expressed RPE65, an RPE marker (Fig. 5q), and others further migrated into the inner retina, became inner retinal cells or exchanged materials with inner retinal cells, including bipolar, ganglion, and glial cells (marked by PKCα, Brn3, and GFAP, respectively, in Fig. 5r–t). Taken together, our results demonstrate that transplanted C-Kit^+^ cells have the potential to integrate into the ONL, although as a small population, and that EGFP (and possibly other molecules) may be transferred to host photoreceptors or to other types of retinal cells. Both activities likely contribute to the population of rescued photoreceptors observed here.

### C-Kit^+^ cells suppress microglial activation and gliosis

**Fig. 6:**
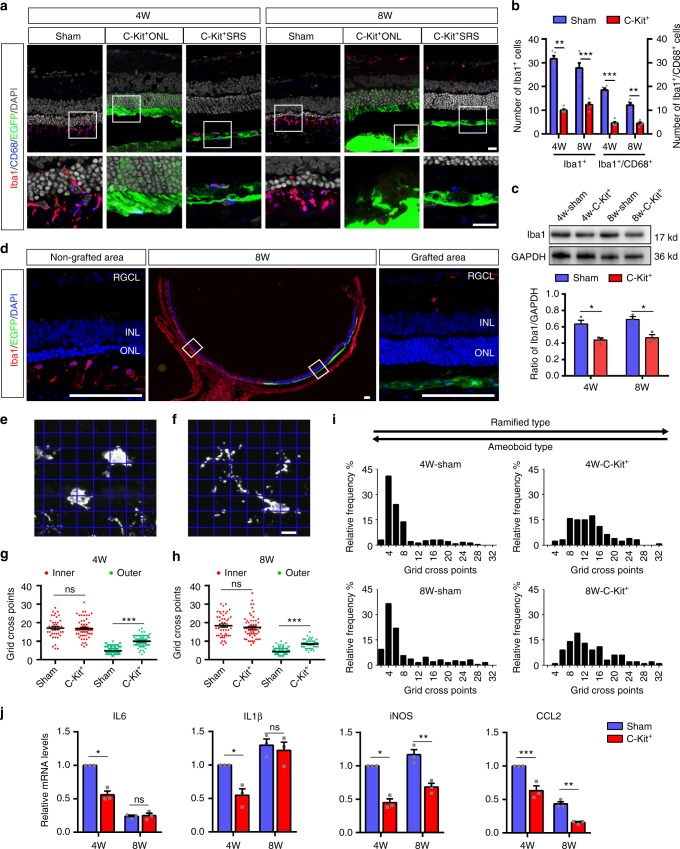
Microglial suppression of RCS rats through grafted C-Kit^+^ cells. **a** Iba1 and CD68 staining in the C-Kit^+^ cell group (showing EGFP^+^ cells extended to the ONL (C-Kit^+^ONL) or only in the SRS (C-Kit^+^SRS)) and the sham group at PO 4w and 8w. **b** Statistical analysis of the number of Iba1^+^ microglial cells and Iba1^+^/CD68^+^phagocytic microglial cells counted in confocal images (*n* = 5 eyes/group). **c** Western blot analysis of Iba1 protein level (*n* = 3 eyes/group). **d** Whole retinal section image showing Iba1 staining in the non-grafted area and the grafted area at PO 8w. **e**, **f** Representative images of ameboid-like (**e**) and ramified microglia (**f**) analyzed by grid crossing. **g**, **h** Statistical analysis of the grid-crossing points in each microglial cell in the inner retina (including RGCL, IPL, INL, and OPL) and the outer retina (including ONL and SRS) of sham and C-Kit^+^ cell-transplanted RCS rats at PO 4w and 8w. **i** Histogram showing the distribution of grid-crossing points per individual microglial cell in retina at PO 4w and 8w. Microglial cells from at least three eyes per group, *n* = 233 cells for 4w-Sham group, *n* = 127 cells for 4w-C-Kit^+^ group, *n* = 192 cells for 8w-Sham group, *n* = 100 cells for 8w-C-Kit^+^ group. **j** Real-time qPCR analysis showing relative mRNA expression for the inflammatory factors IL6, IL1β, iNOS, and CCL2 in the retinas of the C-Kit^+^ and sham groups. (*n* = 3 eyes/group). *P* values were determined by unpaired two-tailed Student’s *t*-test (**b**, **c**, **g**, **h**, **j**) *P < 0.05; **P < 0.01; ***P < 0.001; ns, not significant. Data are presented as mean ± SEM. Scale bars, 20 μm (**a**), 100 μm (**d**), 7 μm (**e**, **f**). GCL ganglion cell layer, IPL inner plexiform layer, INL inner nuclear layer, OPL outer plexiform layer, SRS subretinal space, IL6 interleukin-6, IL1β interleukin-1-beta, iNOS inducible nitric oxide synthase, CCL2 chemokine (C-C motif) ligand 2

Suppression of reactive microglia and regulation of inflammation might be a strategy for improving the outcome of therapeutic transplantation, but this approach usually requires additional reagents or manipulations^30^. Surprisingly, in our study, we found that the number of Iba1^+^ microglial cells was significantly lower in the C-Kit^+^ transplanted group than in the sham group both at PO 4w and PO 8w (Fig. 6a, b). This finding is consistent with the protein expression level of Iba1 examined by Western blotting (Fig. 6c). In fact, in sections across the same retina, areas with spreading C-Kit^+^ cells contained far fewer microglia than the non-grafted areas at PO 8w (Fig. 6d). CD68 is a marker that reflects the phagocytic activity of microglia, which plays a role in photoreceptor death during RD^31^. The number of Iba1^+^/CD68^+^ phagocytic microglia was significantly lower in the C-Kit^+^ group than in the sham group (Fig. 6a, b). Furthermore, using translocator protein (18 kDa, TSPO) as a marker of activated microglia^32^, we found that the C-Kit group had fewer TSPO/CD11b double-labeled cells than the sham group at both PO 4w and 8w, suggesting that fewer activated microglia were present (Supplementary Fig. 5a). The decrease in TSPO in the retinas of the C-Kit^+^ group at PO 4w was confirmed by western blotting (Supplementary Fig. 5b, c). As microglial morphology is a good indicator of the cell’s activation state, we characterized the morphological differences in microglia in different treatment groups using a grid cross-counting system as reported previously^33–35^. Our results revealed that Iba1-positive cells within the outer retinas of C-Kit^+^ cell-transplanted RCS rats (including the ONL and the SRS) had significantly more grid-crossing points, indicating more ramified microglial morphology than that in the sham transplantation group at PO 4w and 8w, while no significant differences in the number of grid-crossing points were found for microglia in the inner retina (Fig. 6e–h). The histogram of the distribution of grid-crossing points demonstrates that the retinal microglia in the C-Kit^+^ group tend to have more ramified shapes (Fig. 6i). Together, the reduced number of activated microglia, the reduced level of activation markers, and the presence of a less active morphology strongly suggest that microglial suppression occurs in C-Kit^+^ cell-transplanted retinas. Interestingly, this effect was not limited to retinal areas with integration and material transfer in the ONL (C-Kit^+^ONL), but also occurred where grafted cells spread extensively across the SRS (C-Kit^+^SRS) (Fig. 6a, Supplementary Fig. 5a).

Reactive microglia often lead to excessive production of inflammatory mediators. As a consequence of cell transplantation, the mRNA expression levels of pro-inflammatory factors, including interleukin-6 (IL6), interleukin-1-beta (IL1β), chemokine (C-C motif) ligand 2 (CCL2), and inducible nitric oxide synthase (iNOS), were significantly decreased in the retinas of the C-Kit^+^ group compared with the sham group at PO 4w (Fig. 6j). A decrease in CCL2 and iNOS mRNA was also observed at PO 8w. Therefore, instead of recruiting and being attacked by microglia/macrophages^19^, C-Kit^+^ cells are able to suppress microglial activation, an ability that is vital to the survival of both transplanted cells and residual host photoreceptors during retinal degeneration.

**Fig. 7:**
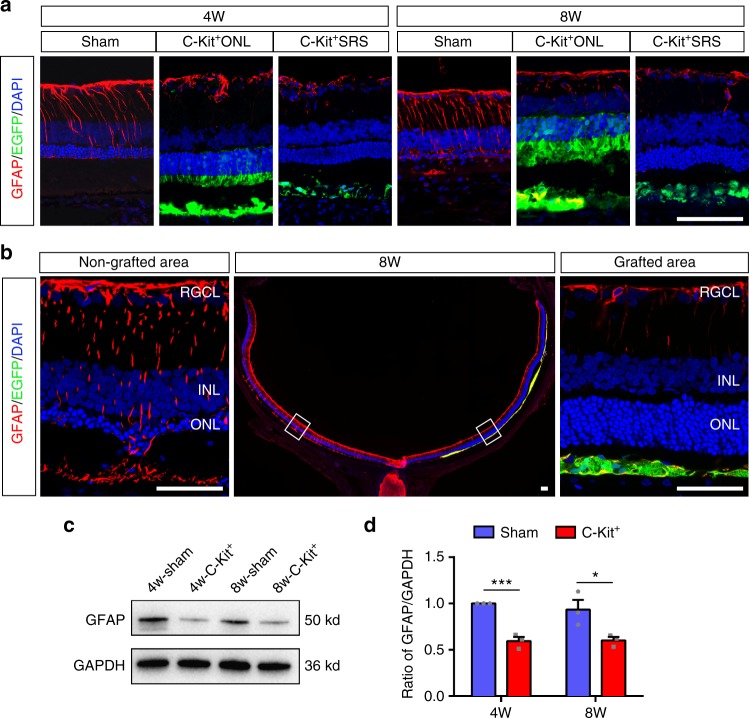
Suppression of gliosis through C-Kit^+^ cells transplantation in RCS rats. **a** GFAP staining in the C-Kit^+^ cell group (both C-Kit^+^ONL and C-Kit^+^SRS) and the sham group at PO 4w and 8w. **b** Whole retinal section image showing GFAP staining in the non-grafted area and the grafted area at PO 8w. **c**, **d** Western blot analysis of GFAP protein level (*n* = 3 eyes/group). *P* values were determined by unpaired two-tailed Student’s *t*-test: *P < 0.05; **P < 0.01; ***P < 0.001. Data are presented as mean ± SEM. Scale bars, 100 μm (**a**, **b**)

Furthermore, the restrained microglial response was accompanied by suppression of gliosis, another pathological change that occurs in RD retinas and impedes grafted cells^36^. GFAP labeling in retinal sections was greatly suppressed in the C-Kit^+^ group compared with the sham group at both PO 4w and PO 8w (Fig. 7a). Similar to the pattern of microglial suppression (Fig. 6d), we observed a remarkable difference in GFAP expression in grafted and non-grafted areas of the same retinas of RCS rats at PO 8w (Fig. 7b). The suppression of gliosis was also indicated by GFAP Western blot analysis (Fig. 7c, d).

**Fig. 8:**
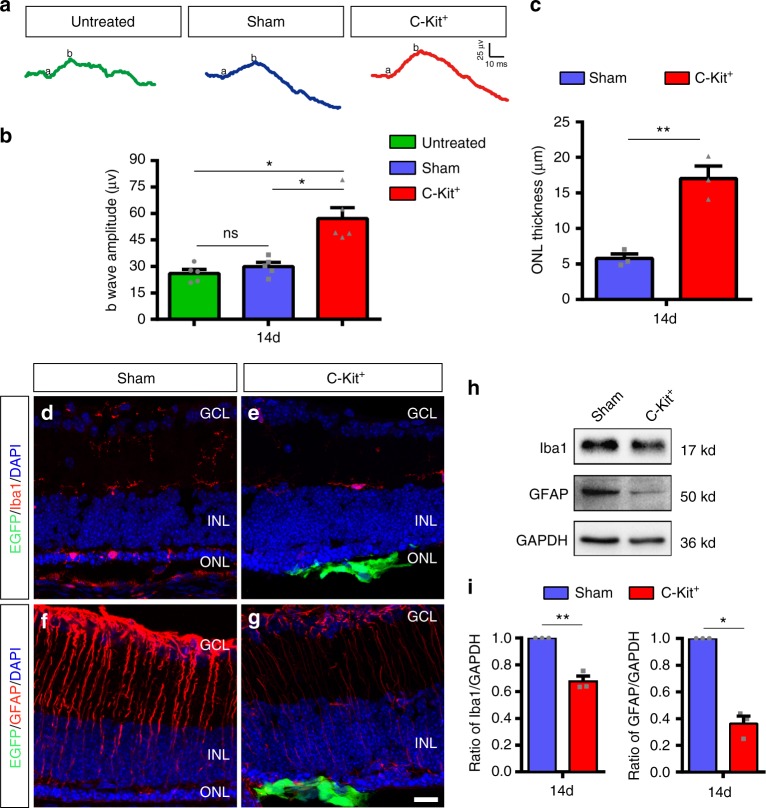
Improvement of retinal function and microenvironment by C-Kit^+^ cell in rd1 mice. **a** Representative b-waves of the C-Kit^+^, sham, and untreated groups of rd1 mice at PO 14d. **b** Statistical analysis of the amplitude of ERG b-waves in the C-Kit^+^, sham, and untreated groups at PO 14d (*n* = 5 eyes/group). **c** Statistical analysis of ONL thickness at PO 14d (*n* = 3 eyes/group). **d**, **e** Iba1 staining at PO 14d. **f**, **g** GFAP staining at PO 14d. Scale bar, 20 μm. **h**, **i** Western blot analysis of Iba1 and GFAP protein levels (*n* = 3 eyes/group). *P* values were determined by one-way ANOVA followed by Dunnett’s T3 multiple comparison tests (**b**) *P < 0.05; **P < 0.01; ***P < 0.001, ns not significant. *P* values were determined by unpaired two-tailed Student’s *t*-test (**c**, **i**) *P < 0.05; **P < 0.01; ***P < 0.001. Data are presented as mean ± SEM

To further corroborate the protective effect of C-Kit^+^ cell transplantation observed in RD models, we conducted similar experiments in rd1 mice, a well-characterized RD model that carries the PDE6B mutation. This mutation causes rapid photoreceptor degeneration that is accompanied by gliosis and microglial activation^37^. Our results showed that transplantation of C-Kit^+^ cells also protected the ONL structure and improved visual function in rd1 mice at PO 14 days (14d) (Fig. 8a–c). In rd1 mice, a similar suppression of microglia and gliosis by grafted C-Kit^+^ cells at PO 14d were also observed (Fig. 8d–i). It suggests that grafted C-Kit cells inhibits microglial activation and gliosis in the degenerative retina.

### C-Kit^+^ cells modulate microglia in vitro

**Fig. 9:**
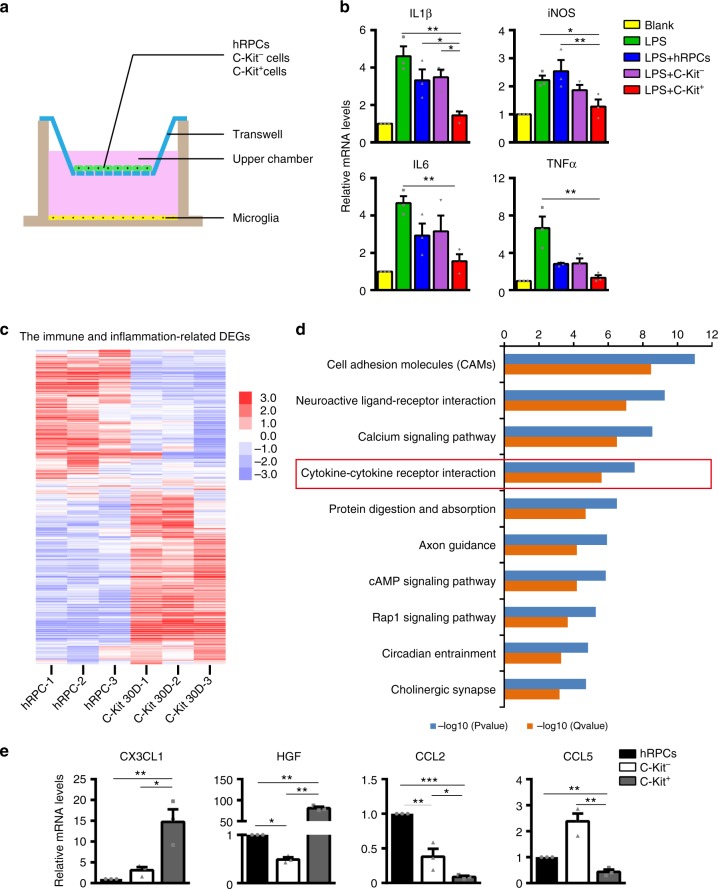
Immune-related transcriptome analysis of C-Kit^+^ cells and primary hRPCs. **a** Diagram of co-culture system. **b** Relative mRNA levels for the inflammatory factors IL1β, iNOS, IL6, and TNFα in LPS-stimulated human microglia cells co-cultured with C-Kit^+^ cells, C-Kit^−^ cells and hRPCs (1 × 10^4^ cells). Human microglia cells were stimulated with 1 μg/ml LPS for 12 h. *n* = 3 independent experiments/group. **c** Heatmap analysis showing differentially expressed immune and inflammation-related genes in C-Kit^+^ cells from 30-day hEROs and hRPCs based on Gene Ontology Analysis (listed in Supplementary Data 2). Log2 expression levels of the genes are shown as a red-white-purple gradient. **d** KEGG pathway analysis of differentially expressed genes showing the top ten pathways associated with genes that are differentially expressed in C-Kit^+^ cells from 30-day hEROs and hRPCs (listed in Supplementary Data 3) (*n* = 3 biologically independent samples/group). **e** Real-time qPCR analysis showing relative mRNA expression for the microglia-suppressing cytokines CX3CL1 and HGF and the microglia/macrophage recruitment-related cytokines CCL2 and CCL5 in C-Kit^+^ cells, C-Kit^−^ cells and hRPCs (*n* = 3 independent experiments/group). *P* values were determined by one-way ANOVA followed by Tukey multiple comparison tests (**b**, **e**) *P < 0.05; **P < 0.01; ***P < 0.001. Data are presented as mean ± SEM. IL1β interleukin-1-beta, iNOS inducible nitric oxide synthase, IL6 interleukin-6, TNFα tumor necrosis factor-alpha, CX3CL1 chemokine (C-X3-C) motif ligand 1, HGF hepatocyte growth factor, CCL2 chemokine (C-C motif) ligand 2, CCL5 chemokine (C-C) motif ligand 5

To explore the effect on microglial regulation of C-Kit^+^ cells, we directly probed their impact on the inflammatory response of microglia using an in vitro co-culture method (Fig. 9a). Bacterial lipopolysaccharides (LPS) can trigger a significant increase in the mRNA levels of pro-inflammatory factors, including IL1β, iNOS, IL6 and tumor necrosis factor-alpha (TNFα), in human microglial cells and BV2 mouse microglial cells when optimized LPS concentrations and treatment times are used (Supplementary Fig. 6a, b). This inflammatory response was significantly dampened when LPS-treated human microglial cells were co-cultured with as few as 1 × 10^4^ C-Kit^+^ cells (Supplementary Fig. 6c, Fig. 9b). Similar results were observed with BV2 mouse microglial cells (Supplementary Fig. 6d). Promisingly, C-Kit^+^ cells appeared to be more effective in suppressing the mRNA levels of some inflammatory factors in microglia than C-Kit^−^ cells or hRPCs (Fig. 9b).

Next, we explored the unique features of C-Kit^+^ cells that enable them to modulate microglia. By comparing the transcriptomes of 30-day C-Kit^+^ cells with those of primary hRPCs, we identified a cohort of immune- and inflammation-related DEGs (Fig. 9c, Supplementary Data 2). Additional Kyoto Encyclopedia of Genes and Genomes (KEGG) pathway analysis demonstrated that the cytokine-cytokine receptor interaction pathway, which is closely associated with microglia and the modulation of inflammation^38^, is among the top differentially regulated pathways between C-Kit^+^ cells and primary hRPCs (Fig. 9d, Supplementary Data 3). Among these DEGs, we identified highly expressed microglia-suppressing cytokines, such as chemokine motif ligand 1 (CX3CL1)^39,40^ and hepatocyte growth factor (HGF)^41^, whereas microglia/macrophage recruitment-related cytokines such as CCL2, and chemokine (C-C motif) ligand 5 (CCL5)^42^ were expressed at much lower levels in C-Kit^+^ cells than in primary hRPCs. We confirmed these transcriptomic analysis results using RT-PCR (Fig. 9e). The results suggest that C-Kit^+^ cells may directly regulate microglia via cytokines that they produce and may thus improve the microenvironment, resulting in better graft and host cell survival when transplanted.

## Discussion

Stem cell therapy brings hope for the treatment of retinal degenerative diseases. However, one critical problem in its clinical application is the lack of optimal donor cells. Here, we developed a surface marker sorting strategy that can be used to acquire a non-tumorigenic population of RPCs from hEROs, which, among other superior features, possess intrinsic microglial suppression ability, leading to a better therapeutic outcome when transplanted into RD models in rats and mice.

To the best of our knowledge, hEROs are a promising cell source for transplantation. However, suitable surface markers that ensure the safety and efficacy of donor cells have not been identified prior to this study. Recently, CD73 was used to isolate PPCs from mESC-derived neural retinas, which have been shown to integrate into the host retina following transplantation^43^. Although CD73 is highly expressed in adult human retina, it is rarely expressed in human fetal retina (from 12 to 19 weeks) and thus is not ideal for clinical applications^43^. Enriching CD73^+^ donor cells from hEROs seems feasible, but the extended culture period required to obtain more mature hEROs would still be challenging. In contrast, C-Kit, a surface marker that is expressed in both fetal and adult retinas, provides a wide time window for sorting.

We found that C-Kit^+^ cells could be harvested at the specific stage of hEROs and that C-Kit^+^ cells enriched from hEROs closely resemble RPCs from human fetal retinas at the transcriptome level. Safety is another critical issue. Previous studies have shown that ESC-derived cells, including RPCs, may form tumors due to the presence of a mixture of undifferentiated ESCs^44,45^. It demonstrated in present study that unsorted cells from hEROs caused abnormal cell proliferation and tumor-like masses when transplanted into the SRS of RCS rats. By using SSEA4 as a negative selection marker to eliminate undifferentiated hESCs or immature daughter cells of early developmental stages, we prevented tumor formation and uncontrolled cell proliferation in our transplantation experiments, demonstrating the excellent safety features of C-Kit^+^ cells for potential clinical application.

Transplanted RPCs and PPCs have been reported to differentiate into photoreceptors and to form synaptic connections with host retinal neurons^46,47^. However, it has been suggested that the so-called integrated cells might arise by cell fusion and material transfer between donor cells and the recipient’s photoreceptors rather than by donor cell integration into the neuronal circuitry of the host retina^27–29^. This process may represent a setback for the replacement of lost photoreceptors but may also suggest a fresh approach to rejuvenating aged or diseased photoreceptors and decelerating RD. Inspiringly, by confirming their human cell origin, we found a small portion (6 ± 2%) of integration of the transplanted C-Kit^+^ cells. Notably, substantial material transfer was also observed in our study, as evidenced by the presence of EGFP^+^ photoreceptors that lacked human mitochondrial (MTCO2) or human nuclear (HuNu) staining. Intriguingly, we observed that while integrated human cells often appeared isolated and tended to be situated in the first or second row of the ONL, cells that had undergone material transfer mostly occurred in clusters that extended through the ONL layers, suggesting a possible networking mechanism. Previously, material transfer was suggested to be a specific property of PPCs, whereas RPCs from mouse embryos were thought to rarely transfer material to host photoreceptors^27^. Nonetheless, we demonstrated that C-Kit^+^ cells, which are also RPCs, do transfer EGFP to host photoreceptors and even potentially to various types of retinal cells, including RPE cells, ganglion cells, bipolar cells, and Müller glial cells. This finding indicates that material transfer is more common than has been previously thought.

However, the factors that determine the relative ratio of integration and material transfer and the relative contributions of the two processes to photoreceptor rescue remain to be elucidated. Interestingly, Waldron et al.^20^ reported that integration or material transfer may occur in an environment-dependent manner. In a degenerative environment with an incomplete external limiting membrane, as many as 20% of grafted cells can integrate into the retinal circuit, whereas in an intact environment few integrations are observed. Thus, approaches to increase integration, including the ability to manipulate donor cells and improve the microenvironment, remain an exciting area of retinal research.

The efficacy of cell transplantation, especially transplantation for an extended period, depends not only on the donor cells but also on the host microenvironment, as shown in a recent study in which both integration and material exchange from transplanted cells were predicted to be influenced by the host environment^20^. In RD, microglia/macrophage infiltration and activation and the associated chronic inflammation are major contributors to host photoreceptor death^31^. Either inadvertently or as part of an active innate immune response, microglia also play a key role in determining the fate of grafted cells^30^. Activated microglia/macrophages can be recruited to attack and eliminate grafted cells^19^. Thus, regulation of microglia is considered a potential therapeutic target for RD or an additional approach in combination with transplantation therapy. Previous work has shown that certain neural stem cells and mesenchymal stem cells can regulate microglia^48–50^. However, to the best of our knowledge, no RPCs have been reported to have intrinsic microglial suppression capability. Intriguingly, SCF/C-Kit signaling has been reported to be involved in microglial modulation^22,23,51^. Additionally, C-Kit^+^ stem cells have been shown to be capable of immunomodulation and microglial suppression in other systems^52–54^. Here, we show that C-Kit^+^ cells, in addition to being RPCs that can be used in transplantation, also possess intrinsic microglia-suppression capability. This finding may explain why we observed relatively long-term graft cell survival and vision preservation. Mechanistically, we suggest that C-Kit^+^ cells have a distinct mRNA expression profile that enhances the release of microglia-suppressing cytokines while inhibiting the production or activity of inflammatory and microglia/macrophage recruitment-related cytokines. For example, C-Kit^+^ cells contain high levels of CX3CL1, a key chemokine that upon interaction with its receptor CX3CR1 attenuates microglial activation and inflammation^39,40^. HGF, which plays an role both in the suppression of microglia and in the protection of neurons^41^, is also drastically upregulated in C-Kit^+^ cells. Gliosis is another pathological change that occurs in RD; it presents a physical barrier and/or chemical inhibition that limits the migration and integration of grafted cells. Perhaps as a consequence of muted microglial activity^55^, we observed an anti-gliosis effect in retinas transplanted with C-Kit^+^ cells; this effect may facilitate the spread of grafted C-Kit^+^ cells through the SRS (Fig. 7) and prevent their accumulation at the injection site.

Notably, the RCS rats that were used in this study provide a classical model of RD that suffers from Mertk mutation-derived RPE dysfunction. This dysfunction subsequently leads to photoreceptor death, remodeling of the inner retina, and substantial gliosis and microglial activation^56^. This complicated form of RD resembles many of the clinical manifestations seen in RD patients and has been widely used in preclinical studies^57,58^. To generalize our findings, we also performed C-Kit^+^ cell transplantation in rd1 mice and observed similar protective effects on retinal morphology and visual function as well as suppression of microglia and gliosis. Although we cannot rule out the possibility of replacement, material exchange, and functional improvement in RPE after transplantation, we attribute this outstanding therapeutic effect to the superior intrinsic properties of C-Kit^+^ cells, especially their ability to suppress microglial activation and gliosis.

In summary, our surface marker sorting strategy is promising for acquiring safe and effective donor cells from hEROs that possess the unique feature of regulating the microenvironment for potential stem cell transplantation therapy in patients with RD.

## Methods

### Animals

RCS rats and rd1 mice, two known models of RD, were provided by the Experimental Animal Center of Third Military Medical University (Army Medical University). All animal work has been approved by the Institutional Review Board of the Third Military Medical University (Army Medical University). The animals, regardless of sex, were raised in a specific pathogen-free room in the Animal Care Centre and maintained under a 12-h light/dark cycle. The animals were allocated randomly for experiments. At least three individuals were used for each experiment.

### Generation of hESC-derived retinal organoids

Human embryonic stem cell line (H9) was kindly provided by Stem Cell Bank, Chinese Academy of Sciences. After being tested without mycoplasma contamination, this hESC line was used in present study and maintained in mTeSR1 (Stem Cell Technologies) without feeders. hEROs were generated following Kuwahara’s protocol^10^. In brief, hESCs were dissociated into single-cell suspensions in TrypLE Express (Gibco) containing 0.05 mg/ml DNaseI (Roche) and 20 μM Y-27632 (Merck). The retinal differentiation medium consisted of 45% IMDM (Gibco), 45% F12-Glutamax (Gibco), 450 μM monothioglycerol (Sigma-Aldrich), and 1% Chemically Defined Lipid Concentrate (Gibco) supplemented with 10% knockout serum replacement (KSR, Gibco) and 20 μM Y-27632. On day 0, 100 μl of cell suspension (1.2 × 10^4^ cells) was placed in each well of low-cell-adhesion V-bottom 96-well plates (Sumitomo Bakelite). On day 6, the medium was exchanged for fresh retinal differentiation medium supplemented with 1.5 nM bone morphogenetic protein 4 (BMP4, Peprotech). Thereafter, half of the medium was replaced every three days. On day 18, hERO was transferred to low-cell-adhesion dishes (Greiner) in long-term culture medium containing Dulbecco’s modified Eagle’s medium (DMEM)/F12-Glutamax (Gibco) with 1% N2 supplement (Gibco), 10% fetal bovine serum (FBS, Gibco), 0.5 μM RA (Sigma) and 0.1 mM taurine (Sigma).

### Flow cytometry

30, 45, and 60-day hEROs were dissociated into single-cell suspensions in TrypLE Express (Gibco). Then, cells at a concentration of 1 × 10^6^/100 μl (a test) were incubated with allophycocyanin (APC)-conjugated anti-human C-Kit antibody (Biolegend, 313206, 5 μl/test), APC Mouse IgG1, κ Isotype Control Antibody (Biolegend, 400120, 5 μl/test), or fluorescein isothiocyanate (FITC)-conjugated anti-human SSEA-4 antibody (BD Biosciences, 560126, 20 μl/test) or FITC Mouse IgG3, κ Isotype Control antibody (BD Biosciences, 555578, 20 μl /test) for 30 min at 4 °C. Cultured C-Kit^+^/SSEA4^−^ cells were fixed with fixation and permeabilization buffer (eBioscience). After blocking with 1% serum, the cell suspension was incubated with Phycoerythrin (PE)-conjugated anti-human Nanog antibody (BD Biosciences, 560483, 20 μl/test) and FITC-conjugated anti-human OCT4 antibody (BD, 560217, 5 μl/test) (1 h, 4 °C). For EGFP label rate detection, P3 EGFP-labeled cells were dissociated into single-cell suspensions and detected with unlabeled cells as a control.

All flow cytometry was performed on BD FACS Aria II and BD FACS Calibur flow cytometer, and data were analyzed with FlowJo software. The gating or sorting strategies for all flow cytometry analysis in this study are shown in Supplementary Fig. 7.

### Culture and retinal differentiation of hEROs-derived RPCs

hEROs-derived RPCs were placed onto vitronectin-coated culture surfaces (Peprotech). Cells are cultured with UltraCULTURE medium (Lonza) supplemented with 10 ng/ml human epidermal growth factor (EGF; Peprotech), 20 ng/ml human basic fibroblast growth factor (bFGF; Peprotech), 1% Glutamax supplement (Gibco), 1% N2 (Gibco), 2% B27 (Gibco) and 20 μM Y-27632 (Merck) for 24 h after isolation from hEROs and incubated at 37 °C in a standard incubator (20% O_2_ or 3% O_2_, 5% CO_2_). The Y27632 medium was changed after 24 h. When passaging, the cells were dissociated into single-cell suspensions using TrypLE Express (Gibco), and the number of cells was recorded. The proliferation of C-Kit^+^ cells was measured based on the cell doubling time (*D*_*T*_) according to the following equation:

$$D_T = t \times \left[ {lg2/\left( {lgN_t - lgN_0} \right)} \right]$$

where *N*_0_ represents the initial cell number and *N*_*t*_ represents the cell number at time period *t*.

For photoreceptor differentiation^16,59^, C-Kit^+^ cells at P3 were resuspended in DMEM/F12 medium (Gibco) containing 1% N2 (Gibco), 2% B27 (Gibco), 2 mM l-glutamine (Gibco), and 10 ng/ml bFGF (Peprotech), seeded on cell slides in 24-well culture plates at a density of 1 × 10^5^ cells/well, and cultured for 4 days. Mature differentiation medium consisting of DMEM/F12 medium (Gibco) containing 2% B27 (Gibco), 10 ng/ml brain-derived neurotrophic factor (BDNF, Peprotech), 10 ng/ml insulin-like growth factor 1(IGF-1, Peprotech), and 10 ng/ml nerve growth factor (NGF, Peprotech) was used for another 4–6 days. For multiple retinal cell differentiation, C-Kit^+^ cells at P3 were cultured in DMEM/F12 medium (Gibco) containing 1% N2 (Gibco), 2mM l-glutamine (Gibco), 10% FBS (Gibco), and 0.5 μM RA (Sigma) for 14 days.

### Teratoma assay

100 μl (1 × 10^7^ cells) of a suspension of hESCs or P3 C-Kit^+^ cells was subcutaneously implanted into the groin area of SCID mice of either sex^16^ (*n* = 6 per group). The condition and the tumor size of the animals were monitored weekly. Three months later, the animals were anesthetized and examined by a pathologist to identify microscopic pathological changes and evidence of teratoma formation.

### Colony-forming efficiency assay

For the colony-forming efficiency assay^17^, 100 P3 C-Kit^+^ cells were plated in a 100-mm-diameter dish (a density of ≈ 2 cells/cm^2^). The culture medium was changed every 2 days. Clones were observed at ~2–3 weeks.

### Isolation and culture of hRPCs

hRPCs were isolated from the retinas of human fetal eyes at 12–14 weeks of gestation^8^; the retinas were provided by the embryonic tissue bank in the Department of Obstetrics in Southwest Hospital, Third Military Medical University (Army Medical University). All experiments involving in human cells and tissues were conducted conforming to the Tenets of the Declaration of Helsinki and this study was approved by the ethics committee of Southwest Hospital, Third Military Medical University (Army Medical University). Briefly, the neural retina was dissociated into small pieces and enzymatically digested into a cell suspension using 1 ml papain (12 units/ml; Worthington). The cells were centrifuged and resuspended in UltraCULTURE medium (Lonza) supplemented with 10 ng/ml EGF (Peprotech), 20 ng/ml bFGF (Peprotech), and 1% Glutamax (Gibco). The culture media was changed every 2 days.

### Transcriptome analysis

For transcriptome analysis, 30, 45, and 60-day isolated C-Kit^+^ cells and hRPCs at P3 were incubated in RNAiso Plus (Takara) at a concentration of 2 × 10^6^ cells/ml and stored at −80 °C. Three experimental replicates per group were performed. All samples were transported to the Genomics Institute on dry ice for the transcriptome study. mRNA was enriched using oligo (dT) magnetic beads and fragmented into short fragments using fragmentation buffer. cDNA libraries were produced and qualified using an Agilent 2100 Bioanalyzer and an ABI StepOnePlus Real-Time PCR System. Primary raw reads produced by HiSeq 4000 (Illumina) were qualified and filtered to obtain clean reads. The Pearson correlation coefficients was based on all gene expression levels. A heatmap analysis of gene expression levels was created based on the averaged fragments per kilobase of exon per million fragments mapped (FPKM) values of genes in 30, 45, and 60-day isolated C-Kit^+^ cells as well as hRPCs. Genes with a fold change in expression >2 and adjusted *P* value ≤ 0.001 were considered to be DEGs^60^. Annotation analysis of Gene Ontology (GO) was performed for screened immune and inflammation-related DEGs. The KEGG database was used to perform pathway analysis of DEGs.

### Cell labeling and subretinal transplantation

hEROs-derived cells or hRPCs were infected with lentivirus carrying CMV promoter-driven EGFP. P3 EGFP-labeled cells were digested with TrypLE Express (Gibco) and resuspended in sterile HBSS (1×, HyClone) supplemented with DNase I (0.005%, Roche) for transplantation. Subretinal transplantation was performed in RCS rats at P21 or in rd1 mice at P7. One eye of RCS rats or rd1 mice was transplanted with hEROs-derived cells or hRPCs, and the other eye was injected with HBSS (HyClone) as sham control. Eyes that did not receive injections were used as untreated controls. A cell suspension (2 × 10^5^ cells in 2 μl of HBSS for RCS rats; 1 × 10^5^ cells in 1 μl for rd1 mice) was injected into the SRS using a 33-gauge Hamilton needle (Hamilton). All animals, including those without injections, received oral cyclosporine A (210 mg/l, Sandoz, Camberley) dissolved in their drinking water from 24 h before transplantation to PO 2w for rats and PO 1w for mice.

### Electroretinogram recording

Corneal scotopic flush electroretinogram recording from both eyes of rats and mice was performed at PO 2w–16w. In brief, after 12-h dark adaptation, rats or mice were anesthetized. The animals’ pupils were dilated using 1% tropicamide. The body temperature of the animals was maintained at 37 °C by placing them on a heating pad to prevent hypothermia. Two active gold electrodes were placed on each cornea as the recording electrodes. The reference and ground electrodes were placed subcutaneously in the mid-frontal areas of the head and tail, respectively. We applied light stimulation at densities of −2.5, −0.5, −0.02, and 0.5 log (cd*s/m^2^) for RCS rats and 0.5 log (cd*s/m^2^) for rd1 mice. The amplitudes of b-waves were recorded and processed using a RETI-Port device (Roland Consult). All procedures were performed in a dark room under dim red safety light.

### Optokinetic head tracking test

The optokinetic head tracking test was performed. In brief, animals were dark-adapted for at least 12 h before being tested. Visual acuities were recorded for both eyes of each animal at PO 4-24w. Experiments were observed and recorded by a single observer who was blind to the experiment grouping. A platform was set in the center of four inward-facing computer monitor screens with an infrared video camera overhead. The rat was placed on the platform and received rotated grating stimuli (12°/s) using a staircase paradigm program written by MATLAB showing spatial frequencies (0.05, 0.075, 0.1, 0.2, 0.3, 0.4, 0.5, and 0.6 cycles/degree) in 100% contrast to measure the visual acuity in both eyes^61^. The head tracing response was driven by clockwise and counter-clockwise rotations to evaluate each eye. The minimum mean luminance of the monitors was 0.20 cd/m^2^, and the maximum mean luminance of the monitors was 50 cd/m^2^. The highest spatial frequency with a response was recorded as the visual acuity of each eye.

### Tissue preparation and immunofluorescence

For hERO tissue preparation, paraffin embedding was performed. In brief, hEROs were fixed in 4% PFA for 6 h at 4 °C. After dehydration in a graded series of ethanol and chloroform, the hEROs were embedded in paraffin. Using a microtome, 5-μm-thick paraffin sections were prepared. Sections across the long axis of hEROs were selected. After deparaffinization and rehydration of the sections, immunofluorescence was performed. For cultured cells, C-Kit^+^ cells were seeded on cell slides at a density of 5 × 10^3^ cells/cm^2^ and fixed in 4% PFA at 4 °C for 15 min. For retina tissue preparation, RCS rats or rd1 mice were euthanized at PO 2–24w by CO_2_ inhalation, and their eyes were removed. The eye cups were separated and fixed in 4% PFA for 2 h at 4 °C. The eye cups were then transferred to 30% glucose solution overnight for dehydration. After embedding in tissue embedding agent, the samples were cut into sections using a cold microtome and attached to glass slides.

For immunofluorescence, the slides were rinsed in 0.01 M phosphate-buffered saline (PBS) and blocked in 0.01 M PBS supplemented with 3% albumin from bovine serum (BSA) and 0.3% Triton X-100 for 30 min at 37 °C. The slides were then incubated with primary antibodies at 4 °C overnight. The following primary antibodies and dilutions have been used in this study: rabbit anti-PAX6 (Abcam, ab5790, 1:500), rabbit anti-ZO-1 (Invitrogen, 402200, 1:500), mouse anti-CHX10 (Santa Cruz, sc-374151, 1:300), rabbit anti-C-Kit (Santa Cruz, sc-5535, 1:200), mouse anti-SSEA4 (Santa Cruz, sc-59368, 1:500), rabbit anti-Ki67 (Abcam, ab66155, 1:500), mouse anti-RAX (Santa Cruz, sc-271889, 1:500), rabbit anti-Recoverin (Abcam, ab101584, 1:500), mouse anti-Rhodopsin (Abcam, ab98887, 1:500), rabbit anti-PKCα (Abcam, ab32376, 1:500), rabbit anti-GS (Abcam, ab73593, 1:500), mouse anti-β tubulin III (Beyotime, AT809-1, 1:500), rabbit anti-Recoverin (EMD Millipore, AB5585, 1:500), rabbit anti-Gnat-1 (Santa Cruz, sc-389, 1:800), mouse anti-Arrestin (Santa Cruz, sc-271466, 1:500), mouse anti-CTBP2 (Santa Cruz, sc-17759, 1:500), rabbit anti-Synaptophysin (Abcam, ab32127, 1:500), mouse anti-MTCO2 (Abcam, ab110258, 1:200), mouse anti-HuNu (Abcam, ab191181, 1:200), mouse anti-Brn3a (Santa Cruz, sc-8429, 1:500), mouse anti-RPE65 (Abcam, ab78036, 1:500), rabbit anti-Iba1 (Wako, 019-19741, 1:500), mouse anti-CD68 (Abcam, ab955, 1:500), rabbit anti-GFAP (Abcam, ab48050, 1:500), rabbit anti-TSPO (Abcam, ab109497, 1:200), mouse anti-CD11b (Abcam, Ab8879, 1:500). On the following day, the slides were incubated with secondary antibodies (Goat anti-mouse IgG Alexa-Fluor-647 (Life technologies, A21236, 1:1000) or Goat anti-rabbit IgG Alexa-Fluor-568 (Life technologies, A11011, 1:1000) or Goat anti-rabbit IgG Alexa-Fluor-488 (Life technologies, A11001, 1:1000) for 2 h at room temperature. After counterstaining with DAPI, the slides were viewed and photographed using a confocal laser scanning microscope (Leica SP8).

### Western blot analysis

Retinal hemispheres containing the injection area were isolated from RCS rats or rd1 mice at PO 2w, 4w, and 8w. Tissue lysis buffer containing 10% PMSF and 90% RIPA was added to extract protein. Proteins (30 μg per well) were separated on a 12% sodium dodecyl sulfate polyacrylamide gel (SDS-PAGE) and transferred to polyvinylidene fluoride (PVDF) membranes. After blocking with 5% BSA for 1 h at 37 °C, the membranes were incubated with primary antibodies, including rabbit anti-Iba1 (Wako, 016-20001, 1:1000), rabbit anti-GFAP (Abcam, ab48050, 1:2000), mouse anti-GAPDH (Abcam, ab8245, 1:2000), and goat anti-TSPO (Aviva Systems Biology, OALA05219, 1:1000), overnight at 4 °C. The following day, the membranes were washed and incubated with goat anti-rabbit IgG secondary antibody (Invitrogen, #31460, 1:2000) or goat anti-mouse IgG secondary antibody (Invitrogen, 62-6520, 1:2000) or rabbit anti-goat IgG secondary antibody (Invitrogen, 81-1620, 1:2000) for 2 h at room temperature. Finally, the proteins on the membranes were detected using a Pierce™ ECL Western Blotting Substrate (Thermo, 32106) and scanned using a Bio-Rad exposure system (Bio-Rad). Relative protein expression levels were quantified using ImageJ software (NIH) with GAPDH as control. Uncropped images of western blotting and gel are shown in Supplementary Fig. 8.

### ONL thickness analysis

Standard photomicrographs were obtained in five areas of one retinal section that was stained with DAPI. Three sections were chosen from each eye, and at least three eyes were included in each of the C-Kit^+^ cell, sham, and untreated groups. For each section, areas in the middle of the grafted sites and the corresponding grafted areas of sham group were selected. The ONL thickness was calculated based on its vertical length measured using ImageJ.

### Analysis of microglia

Three 40× field views were captured from the corresponding grafted areas of three 15-μm thick retinal sections per eye in the C-Kit^+^ cells group and the sham group using a Leica confocal imaging system with 1-µm *z*-steps. Maximum intensity projections (MIPs) were processed. Subsequently, the morphology of the microglia in the sections was analyzed using a grid system to determine the number of grid-crossing points per individual cell (from at least three different eyes per group) as previously reported^34,35^. The number of grid-crossing points per cell and the relative frequency of crossing were analyzed. The numbers of Iba1^+^ cells and Iba1^+^/CD68^+^ cells were quantified by counting the cells in sections of retinas (*n* = 5 eyes per group).

### Co-culture with human microglia or BV2 mouse microglia

The immortalized human microglia-SV40 cell line derived from primary human microglia was purchased from Applied Biological Materials, Inc. (ABM, T0251) and cultured in high-glucose DMEM supplemented with 10% FBS (Hyclone). Lipopolysaccharides (LPS) (Sigma) at various concentrations (10–2000 ng/ml) were applied for 6–24 h to stimulate human microglia^62^. BV2 mouse microglia was gifted from Dr. Guo of the Neurological Surgery Department of Southwest Hospital^50^ and cultured in high-glucose DMEM supplemented with 10% FBS. LPS at various concentrations (10–2000 ng/ml) was applied for 4 h to stimulate BV2 microglia^63^. The two cell lines were tested for mycoplasma contamination before experiments and they were negative.

For co-culture, microglia was cultured in six-well plates at a density of 1 × 10^5^ cells/well for 24 h and treated with optimized LPS treatment (1 μg/ml, 12 h for human microglia and 1 μg/ml, 4 h for BV2 microglia). The LPS-treated cells were co-cultured with C-Kit^+^ cells, C-Kit^-^ cells and hRPCs seeded on six-well cell culture inserts (Millipore) at a density of 1–100 × 10^3^ per well for 24 h. Monocultures of microglia were processed as a blank control. All of the cultures were maintained under consistent and equivalent conditions.

### RNA isolation and real-time quantitative PCR

Total RNA was extracted from cells and tissues using RNAiso Plus (Takara) followed by chloroform extraction. cDNA was reverse transcribed using a PrimeScript™ RT reagent kit with gDNA Eraser (Takara) according to the manufacturer’s instructions. Real-time qPCR was performed in a CFX96 Real-time PCR System (Bio-Rad) using SYBR® Premix Ex Taq™ II (Takara) to measure the expression of various genes, and the results were normalized to β-actin levels. The primers used are listed in Supplementary Data 4.

### Statistics

Each result is produced from at least three biological samples. Statistical Product and Service Solutions software V22.0 (SPSS) was used for statistical analyses. Data are presented as the mean ± SD or mean ± SEM as indicated in corresponding figure legends. For multiple comparison among groups, one-way analysis of variance (ANOVA) followed by Tukey multiple comparison tests (equal variances) or Dunnett’s T3 multiple comparison tests (unequal variances) were performed. For comparison between two groups, the unpaired two-tailed Student’s *t*-test was performed. Differences were accepted as significant at *P* < 0.05.

### Reporting summary

Further information on experimental design is available in the Nature Research Reporting Summary linked to this article.

## Supplementary information

Supplementary Information

Description of Additional Supplementary Files

Supplementary Data 1

Supplementary Data 2

Supplementary Data 3

Supplementary Data 4

Reporting Summary

## Data Availability

The raw data that support the findings of this study are available from the corresponding author upon reasonable request. The data of transcriptome analysis in this study has been deposited in the NCBI sequence read archive database under accession code PRJNA506329.
