# Supplementary material for: Organoid-derived C-Kit+/SSEA4− human retinal progenitor cells promote a protective retinal microenvironment during transplantation in rodents
Source: Nat Commun. 2019 Mar 14;10:1205. doi: 10.1038/s41467-019-08961-0 (PMC6418223; doi:10.1038/s41467-019-08961-0)
Supplement: Supplementary file 1 — Supplementary Information [file 41467_2019_8961_MOESM1_ESM.pdf]

Organoid-derived C-Kit^{+}/SSEA4^{-} human retinal progenitor cells promote a protective retinal microenvironment during transplantation in rodents

Zou et al.

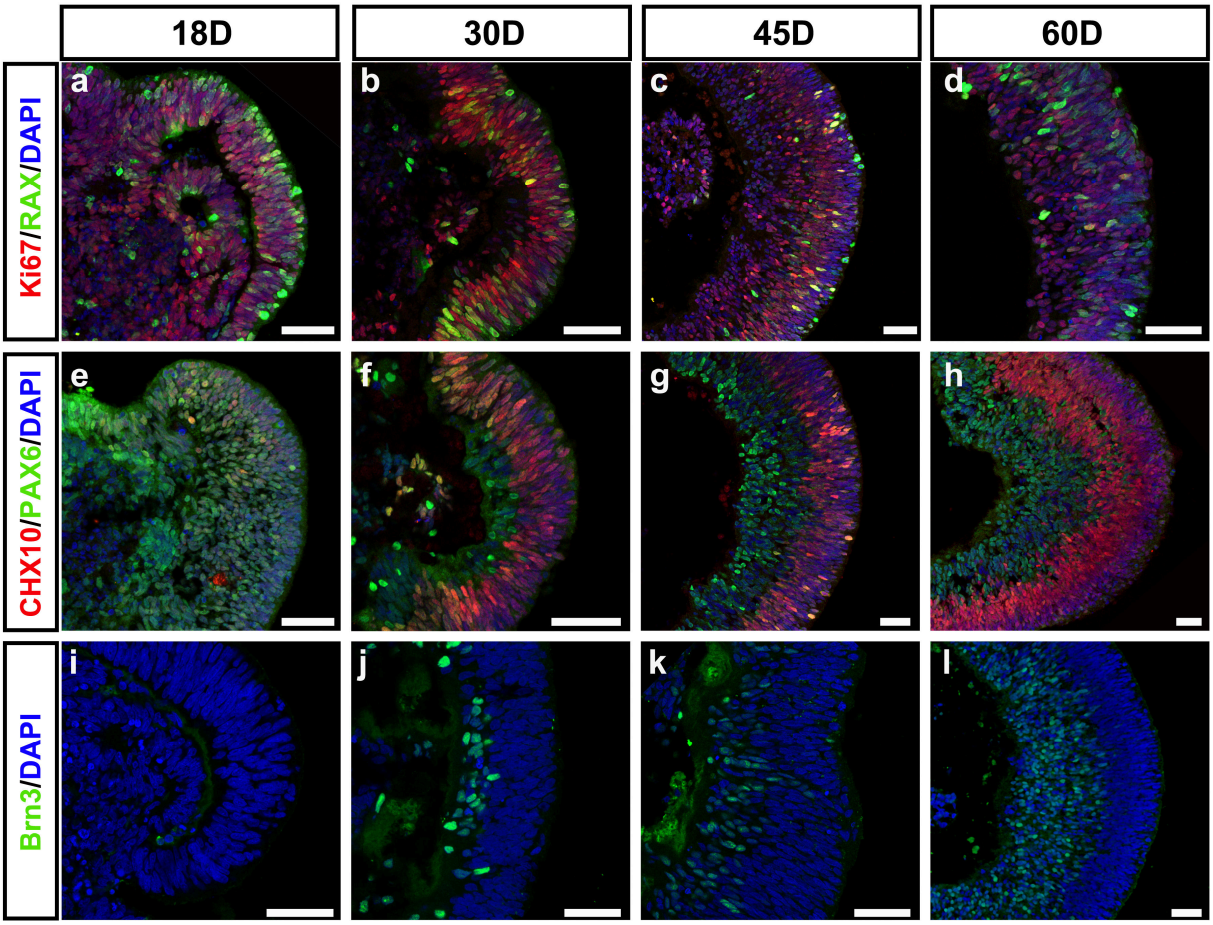

Supplementary Figure 1. Identification of hEROs. a-h: The RPC markers RAX, PAX6, and CHX10 and the proliferation marker Ki67 were labeled in sections obtained from 18-, 30-, 45-, and 60-day hEROs. **i-l:** Brn3a$^+$ ganglion cells were observed at the basal side of the neural retina in sections from 30-, 45-, and 60-day hEROs, and the number of Brn3a$^+$ cells progressively increased with time. Scale bars, 50 µm (**a-l**).

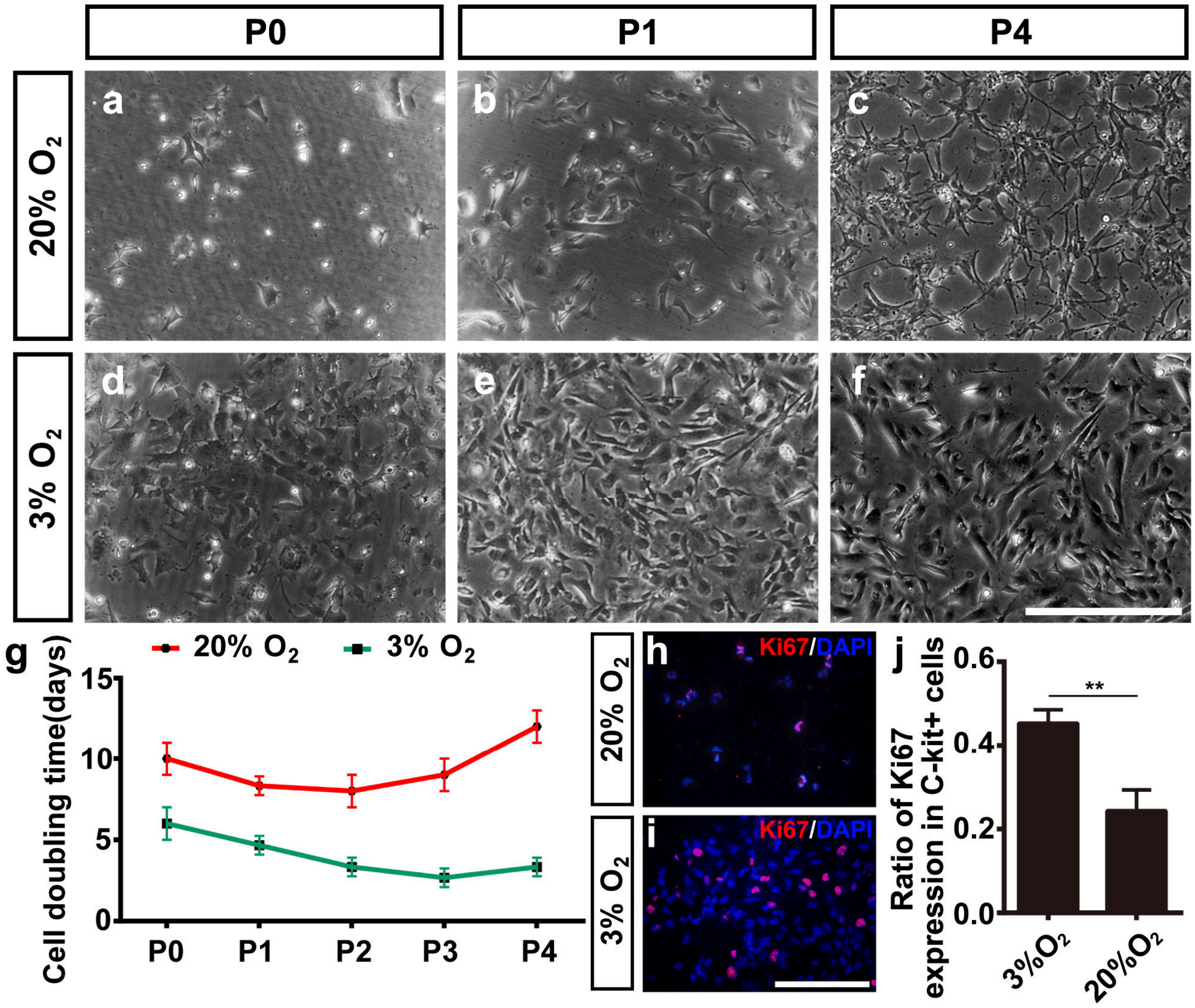

Supplementary Figure 2. Cell proliferation of C-Kit^{+} cells under hypoxic and normoxic conditions. a-f: Representative images of C-Kit^{+} cells maintained in 20% O_2 and 3% O_2 environments at passages 0, 1 and 4 (P0, P1 and P4). **g:** Corresponding statistical analysis of cell proliferation time of C-Kit^{+} cells maintained in 20% O_2 and 3% O_2 at P0 to P4 (n=3 independent experiments/group). **h-j:** Ki67 staining and statistical analysis of C-Kit^{+} cells maintained in 20% O_2 and 3% O_2 (n=3 independent experiments/group). *P* value was determined by unpaired two-tailed Student's *t*-test (**j**): * $P<0.05$; ** $P<0.01$; *** $P<0.001$; ns, not significant. Data are presented as mean ± SEM. Scale bars, 50 μm (**a-f**), 100 μm (**h, i**).

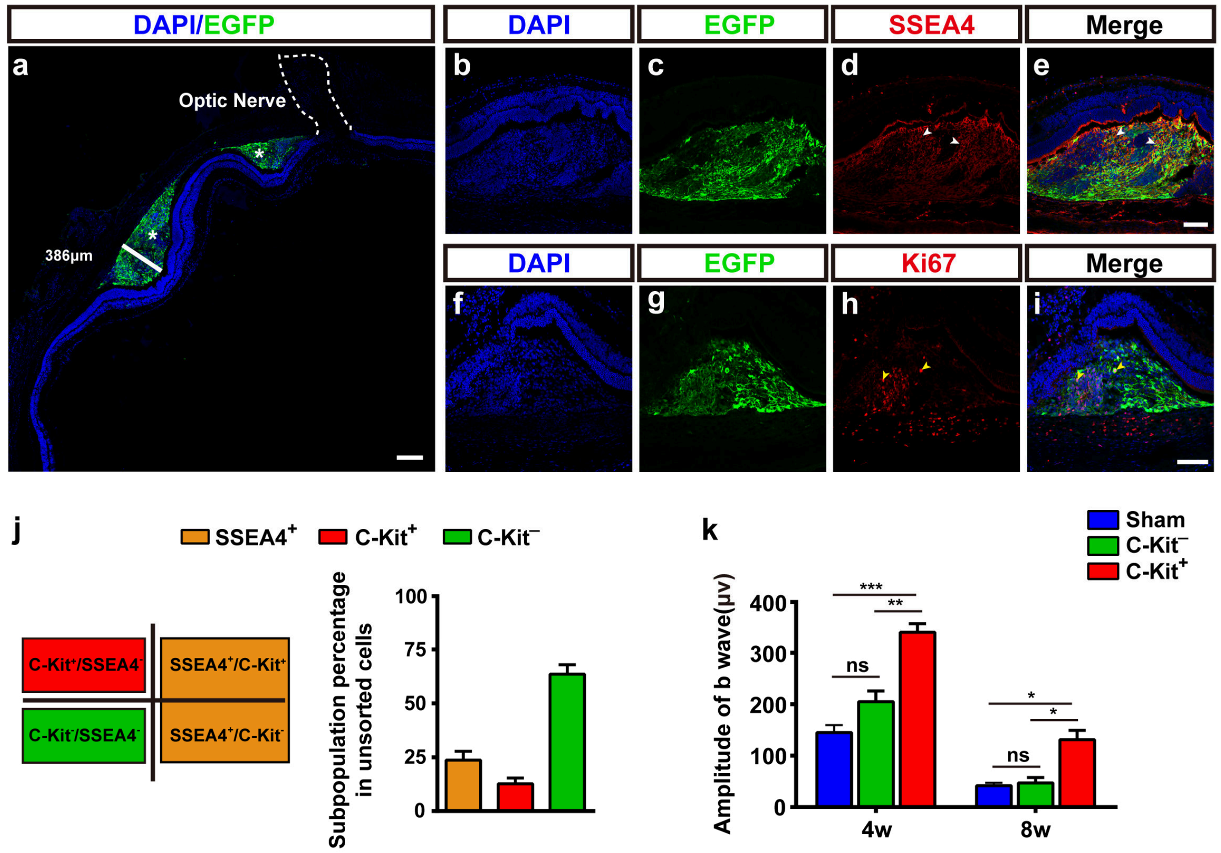

Supplementary Figure 3. Safety and efficacy in transplantation of unsorted cells and subpopulations isolated from hEROs. a: Tumor-like mass and cell proliferation observed in RCS rat subretinal space at PO 4w following transplantation of P3 unsorted 30-day hEROs-isolated cells (white asterisks) (n=4 of 6 eyes). **b-e:** SSEA4 staining showing a cluster of EGFP$^+$ cells co-stained with SSEA4 in the cell mass (white arrowheads). **f-i:** Ki67 staining of cell mass (yellow arrowheads). **j:** Flow cytometry analysis showing the percentage of SSEA4$^+$ cells (SSEA4$^+$/C-Kit$^\pm$ cells), C-Kit$^+$ cells (C-Kit$^+$/SSEA4$^-$ cells), and C-Kit$^-$ cells (C-Kit$^-$/SSEA4$^-$ cells) in P3 unsorted cells from 30-day hEROs (SSEA4$^+$: 23.7±2.3%; C-Kit$^+$ cells: 12.7±1.6%; C-Kit$^-$ cells: 63.6±2.5%; n=3 independent experiments/group). **k:** Statistical analysis of the amplitude of b-waves in the C-Kit$^+$ cells, C-Kit$^-$ cells and sham transplantation groups obtained through fERG (0.5 log(cd*s/m^2)) at PO 4w and 8w (n=5 eyes/group). *P* value was determined by one-way analysis of variance (ANOVA) followed by Tukey multiple comparison tests: * P<0.05; ** P<0.01; *** P<0.001; ns, not significant. Data are presented as mean ± SD. Scale bars, 200 μm (**a**)**,** 100 μm (**b-i**).

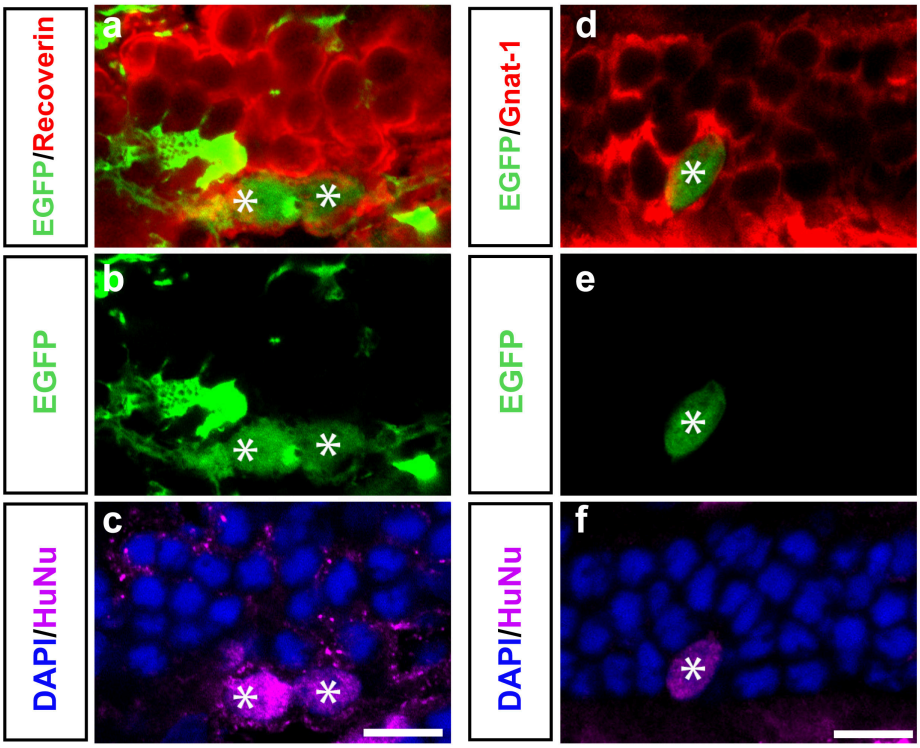

Supplementary Figure 4. EGFP$^+$ grafted cells co-express the human-specific marker and photoreceptor markers. a: EGFP$^+$ cells co-expressed the photoreceptor marker Recoverin. (white asterisks). **b, c:** HuNu staining identifying the EGFP$^+$ cells of human origin (white asterisks). **d:** EGFP$^+$ cells co-expressed the photoreceptor marker Gnat-1 (white asterisk). **e, f:** HuNu staining identifying the EGFP$^+$ cells of human origin (white asterisk). Scale bar: 10μm.

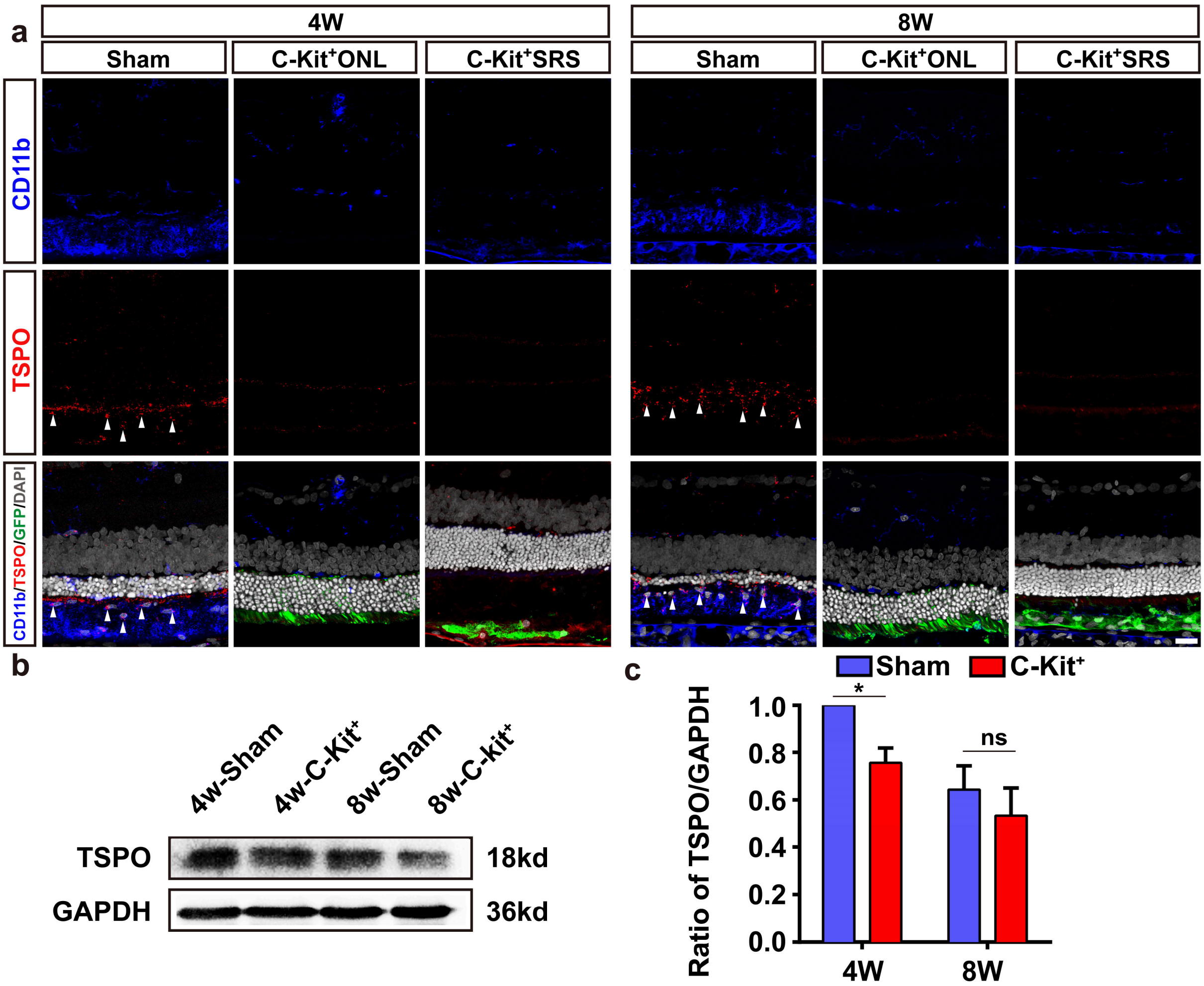

Supplementary Figure 5. TSPO suppression in activated microglia by grafted C-Kit$^+$ cells in the retinas of RCS rats. a: Activated microglia shown by TSPO and CD11b co-staining in the retinas of sham and C-Kit$^+$ cell transplantation groups at PO 4w and 8w (white arrowheads). Scale bar: 20μm. **b, c**: Western blot analysis of TSPO protein levels in retinas of the sham and C-Kit$^+$ cell groups at PO 4w and 8w (n=3 eyes/group). *P* value was determined by unpaired two-tailed Student's *t*-test: * P<0.05; ** P<0.01; *** P<0.001; ns, not significant. Data are presented as mean ± SEM.

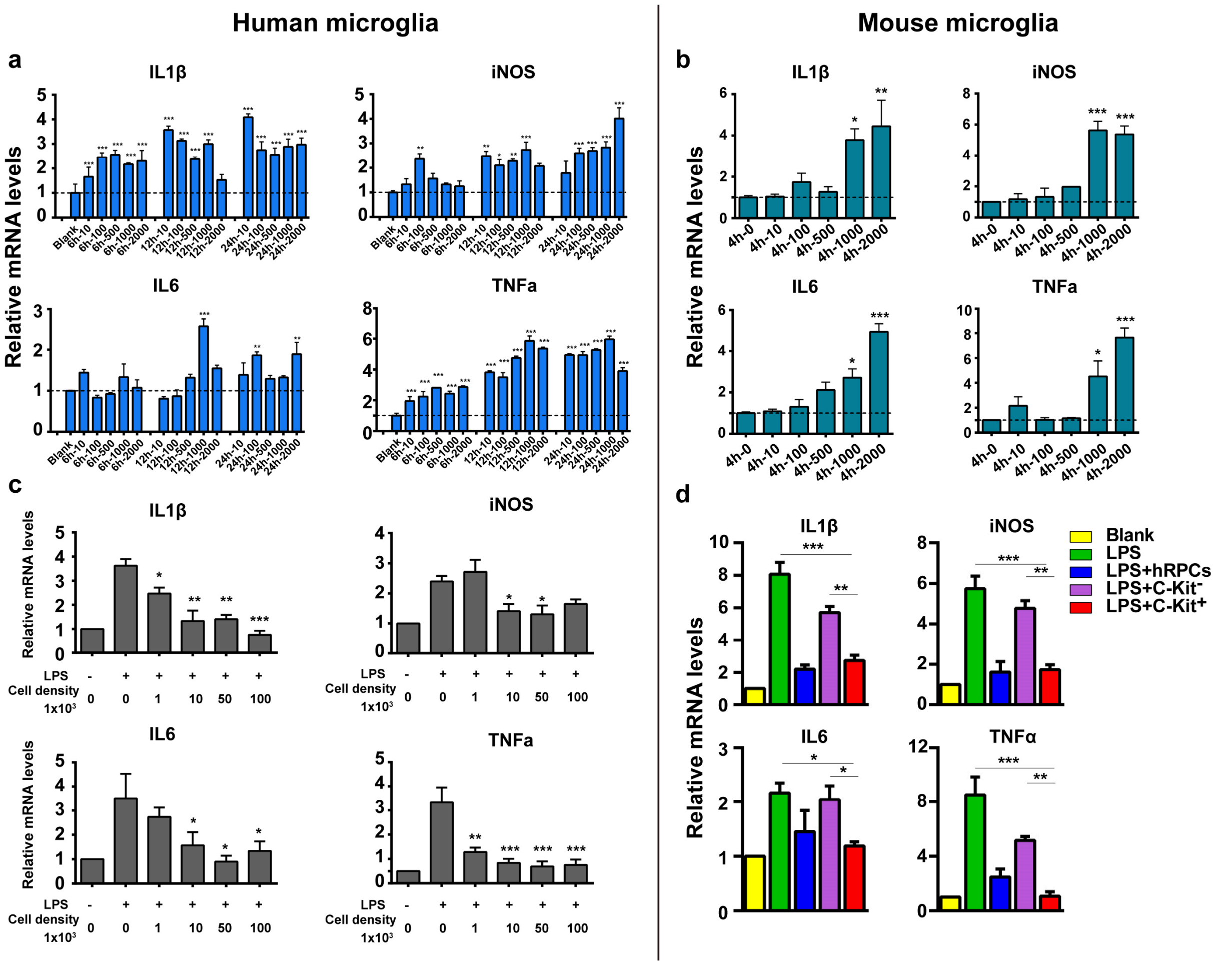

Supplementary Figure 6. Suppression of the inflammatory response by co-cultured C-Kit$^+$ cells in different microglial cells. a: Relative mRNA expression of the inflammatory factors IL1β, iNOS, IL6 and TNFα in an LPS-stimulated human microglial cell line. n=3 independent experiments/group. **b:** Relative mRNA expression of inflammatory factors in LPS-stimulated BV2 mouse microglial cells. n=3 independent experiments/group. **c**: Relative mRNA levels of inflammatory factors in LPS-stimulated human microglial cells co-cultured with C-Kit$^+$ cells at graded cell densities. n=3 independent experiments/group. **d:** Relative mRNA levels of inflammatory factors in LPS-stimulated BV2 mouse microglial cells co-cultured with hRPCs, C-Kit$^+$ cells and C-Kit$^-$ cells. n=3 independent experiments/group. *P* values were determined by one-way analysis of variance (ANOVA) followed by Dunnett's multiple comparison tests (**a-c**): * *P*<0.05; ** *P*<0.01; *** *P*<0.001. *P* values were determined by ANOVA followed by Tukey multiple comparison tests (**d**): * *P*<0.05; ** *P*<0.01; *** *P*<0.001.Data are presented as mean ± SEM.

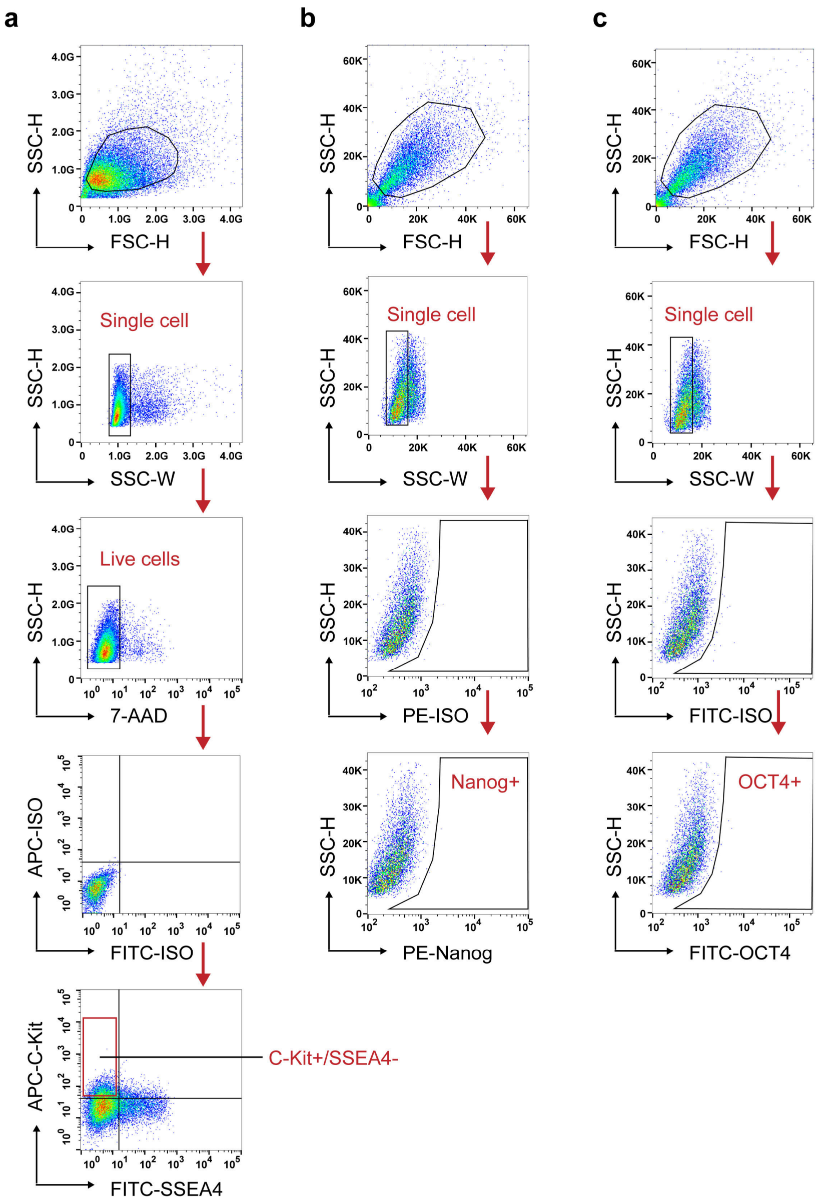

Supplementary Figure 7. The sequential gating or sorting strategies for flow cytometry.

a: The gating strategy for Fig. 1l. **b:** The gating strategy for Fig. 3n. **c:** The gating strategy for Fig. 3o.

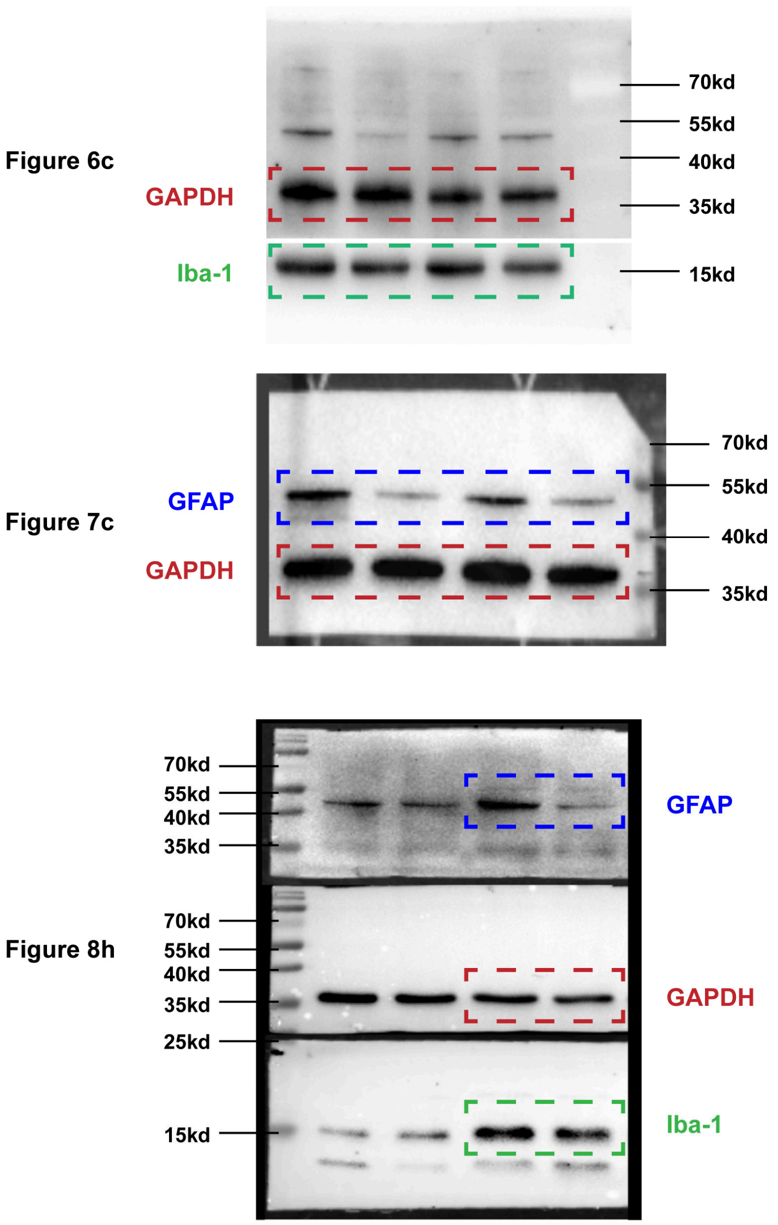

Supplementary Figure 8. Uncropped images of Western blotting and gel in Figure 6-8.
