# Supplementary material for: Organoid-derived C-Kit+/SSEA4− human retinal progenitor cells promote a protective retinal microenvironment during transplantation in rodents
Source: Nat Commun. 2019 Mar 14;10:1205. doi: 10.1038/s41467-019-08961-0 (PMC6418223; doi:10.1038/s41467-019-08961-0)
Supplement: Supplementary file 2 — Description of Additional Supplementary Files [file 41467_2019_8961_MOESM2_ESM.pdf]

Description of Additional Supplementary Files

Supplementary Data 1.
Averaged fragments per kilobase of exon per million fragments mapped (FPKM) value of genes in 30D, 45D, and 60D C-Kit$^+$ cells as well as hRPCs.

Supplementary Data 2.
List of immune and inflammation-related genes differentially expressed between 30D C-Kit$^+$ cells and hRPCs.

Supplementary Data 3.
KEGG pathway analysis of differentially expressed genes between 30D C-Kit$^+$ cells and hRPCs.

Supplementary Data 4.
Primers used in RT-PCR.
