# Supplementary material for: Organoid-derived C-Kit+/SSEA4− human retinal progenitor cells promote a protective retinal microenvironment during transplantation in rodents
Source: Nat Commun. 2019 Mar 14;10:1205. doi: 10.1038/s41467-019-08961-0 (PMC6418223; doi:10.1038/s41467-019-08961-0)
Supplement: Supplementary file 6 — Supplementary Data 4 [file 41467_2019_8961_MOESM6_ESM.docx]

**Supplementary Data 4.** Primers used in RT-PCR

| **Rat primers** | | |
| --- | --- | --- |
| **Genes** | **Forward** | **Reverse** |
| IL6 | CCTGGAGTTTGTGAAGAACAACT | GGAAGTTGGGGTAGGAAGGA |
| IL1β | AGGCTTCCTTGTGCAAGTGT | TGAGTGACACTGCCTTCCTG |
| iNOS | CTTTGTGCGGAGTGTCAGTGG | TTCTTCCTGATAGAGGTGGTCC |
| CCL2 | GCTGTAGTATTTGTCACCAAGCTCAA | GTACTTCTGGACCCATTCCTTATTG |
| β-Actin | ACAACCTTCTTGCAGCTCCTC | CTGACCCATACCCACCATCAC |
| **Mouse primers** | | |
| TNFα | AGCCCACGTCGTAGCAAACCAC | AGGTACAACCCATCGGCTGGCA |
| IL6 | TAGTCCTTCCTACCCCAATTTCC | TTGGTCCTTAGCCACTCCTTC |
| IL1β | CCTGCAGCTGGAGAGTGTGGAT | TGTGCTCTGCTTGTGAGGTGCT |
| iNOS | CCTTGTTCAGCTACGCCTTC | AAGGCCAAACACAGCATACC |
| β-Actin | TGAGCTGCGTTTTACACCCT | TTTGGGGGATGTTTGCTCCA |
| **Human primers** | | |
| CX3CL1 | CTGGCTGCTTGGAGACGAG | GTGCCGCCATTTCGAGTTAG |
| HGF | GAGGGGGCTGGAAGAGAGTA | TGCCTAAAAGAGCCAGTCGG |
| CCL2 | TTCCCCTAGCTTTCCCCAGA | TCCCAGGGGTAGAACTGTGG |
| CCL5 | TGCCCACATCAAGGAGTATTTC | CCATCCTAGCTCATCTCCAAAG |
| TNFα | TCCTTCAGACACCCTCAACC | AGGCCCCAGTTTGAATTCTT |
| IL6 | GAGAGTAGTGAGGAACAAGCCA | TCTTTGAGCCTGTCTTCCCC |
| IL1β | GCTGAGGAAGATGCTGGTTC | TCCATATCCTGTCCCTGGAG |
| iNOS | CGCATGACCTTGGTGTTTGG | CATAGACCTTGGGCTTGCCA |
| β-Actin | ACTCTTCCAGCCTTCCTTC | ATCTCCTTCTGCATCCTGTC |
